# Imaging of congenital lung diseases presenting in the adulthood: a pictorial review

**DOI:** 10.1186/s13244-021-01095-2

**Published:** 2021-10-30

**Authors:** Gamze Durhan, Selin Ardali Duzgun, Meltem Gülsün Akpınar, Figen Demirkazık, Orhan Macit Arıyürek

**Affiliations:** Department of Radiology, Faculty of Medicine, Hacettepe University, 06410 Ankara, Turkey

**Keywords:** Congenital lung diseases, Adults, Tomography (X-ray computed), Magnetic resonance imaging

## Abstract

Congenital lung diseases in adults are rare diseases that can present with symptoms or be detected incidentally. Familiarity with the imaging features of different types of congenital lung diseases helps both in correct diagnosis and management of these diseases. Congenital lung diseases in adults are classified into three main categories as bronchopulmonary anomalies, vascular anomalies, and combined bronchopulmonary and vascular anomalies. Contrast-enhanced computed tomography, especially 3D reconstructions, CT, or MR angiography, can show vascular anomalies in detail. The tracheobronchial tree, parenchymal changes, and possible complications can also be defined on chest CT, and new applications such as quantitative 3D reconstruction CT images, dual-energy CT (DECT) can be helpful in imaging parenchymal changes. In addition to the morphological assessment of the lungs, novel MRI techniques such as ultra-short echo time (UTE), arterial spin labeling (ASL), and phase-resolved functional lung (PREFUL) can provide functional information. This pictorial review aims to comprehensively define the radiological characteristics of each congenital lung disease in adults and to highlight differential diagnoses and possible complications of these diseases.

## Key points

- Congenital lung diseases in adults can be detected incidentally or present with non-specific symptoms such as pneumonia, dyspnea, dysphagia, and pulmonary hypertension.
- Congenital lung diseases can be classified as bronchopulmonary anomalies, vascular anomalies, and combined bronchopulmonary and vascular anomalies.
- Imaging plays a vital role both in the diagnosis of congenital lung diseases and in identifying complications.
- In addition to conventional CT and MRI images, multiplanar, 3D volume-rendered, quantitative 3D reconstruction, and functional MRI images can help in the detection of congenital lung diseases.

## Introduction

**Fig. 1:**
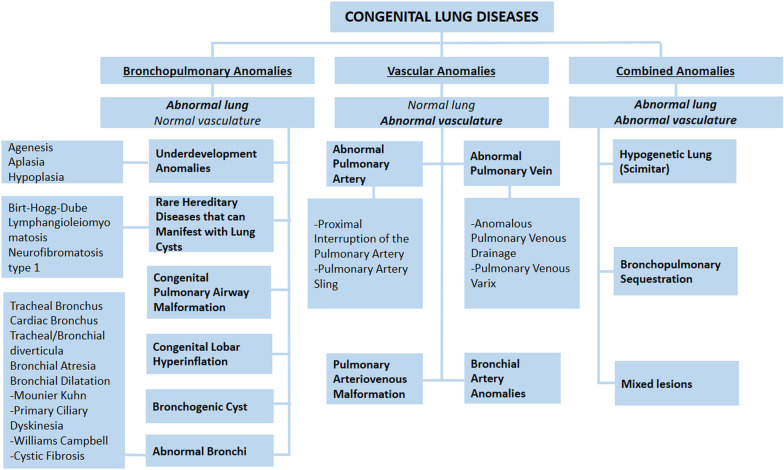
Classification of congenital lung diseases

Congenital lung diseases are rare and are usually diagnosed in childhood. Adults with a history of treated lung malformation in childhood may present for investigation of residual damage in the lung. However, in some patients, congenital lung diseases remain asymptomatic and may be discovered incidentally in adulthood. They can be confused with other lung pathologies, although they may have distinctive radiological findings [1–4]. Familiarity with the imaging findings of different types of congenital lung diseases facilitates both the diagnosis and management of these diseases. Furthermore, the correct diagnosis can prevent potential complications and unnecessary worry of patients as congenital lung abnormalities may mimic neoplasms in adults. Congenital lung diseases in adults can be classified into three main categories: bronchopulmonary anomalies, vascular anomalies, and combined bronchopulmonary and vascular anomalies [1, 4] (Fig. 1). Contrast-enhanced computed tomography (CT) including three-dimensional (3D) or multi-planar reconstruction (MPR) images and magnetic resonance imaging (MRI) can be used reliably as they show the vascular and tracheobronchial structures in detail. Quantitative 3D reconstruction CT images can help image parenchymal changes based on lung density (Hounsfield Unit). In addition to the morphological assessment of the lungs, novel MRI techniques such as ultra-short echo time (UTE), arterial spin labeling (ASL), and phase-resolved functional lung (PREFUL) can provide functional information. This pictorial review aims to comprehensively define the radiological characteristics of each congenital lung disease in adults and highlight differential diagnoses and the potential complications of these diseases.

## Bronchopulmonary anomalies

### Underdevelopment anomalies

Underdevelopment is classified into three types as agenesis, aplasia, and hypoplasia. In pulmonary agenesis, pulmonary parenchyma, bronchus, and vascular structures are completely absent. In pulmonary aplasia, a short rudimentary bronchus is seen without pulmonary vasculature and lung parenchyma. In addition to airways, alveoli, and vascular structures are present, at decreased size and number in pulmonary hypoplasia [4].

Bilateral pulmonary agenesis is a fatal condition causing death in the intrauterine period or within the first hours after delivery. However, pulmonologists, as well as radiologists, may encounter patients with unilateral pulmonary agenesis as the long-term survival rate is approximately 60%. Higher mortality can be related to other congenital anomalies including cardiac malformations [5].

**Fig. 2:**
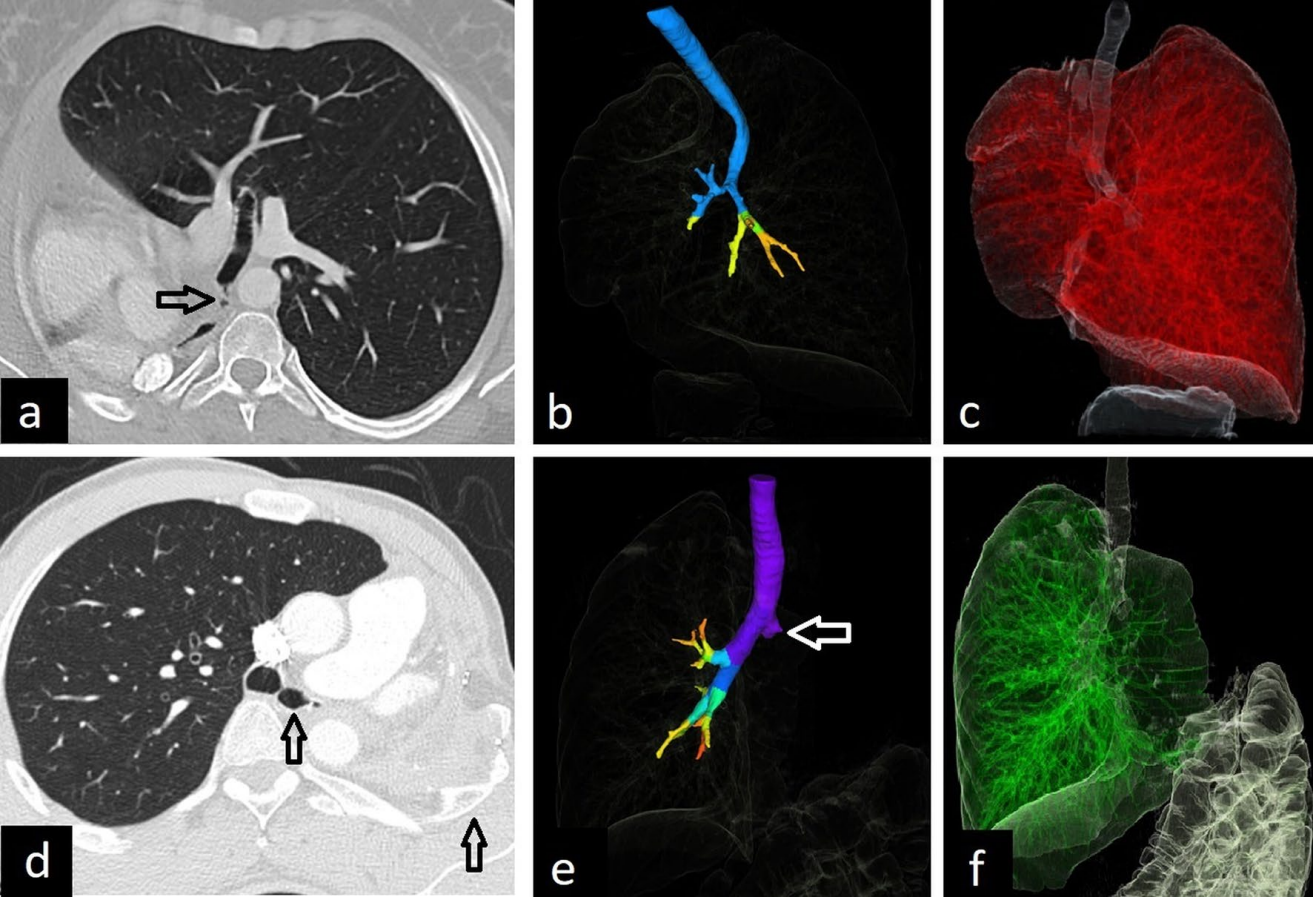
CT images of pulmonary aplasia and pneumonectomy. Rudimentary blind-ending bronchus can be seen on axial CT image in a 27-year-old asymptomatic female with pulmonary aplasia and helps in the differentiation from pulmonary agenesis (**a**) (arrow). 3D reformatted images (**b**, **c**) show the only left airways and compensatory hyperplasia of the left lung into the right side. The left main bronchial stump is observed on axial CT and 3D reformatted images in a 33-year-old patient with right pneumonectomy (**d**–**f**) (arrows). Rib deformities and pleural fluid due to pneumonectomy are depicted on axial CT image (**d**) (arrow)

Pulmonary underdevelopment causes compensatory hyperplasia of the remaining lung. Pulmonary agenesis and aplasia have similar imaging findings and simulate pneumonectomy. Contralateral lung herniation across the midline and prominent ipsilateral mediastinal shift are characteristics imaging features. Opacified hemithorax with mediastinal shift on frontal chest X-ray and hyperlucency in the anterior chest due to herniation of contralateral hyperinflated lung on lateral chest X-ray can be seen. Pulmonary aplasia can be distinguished from pulmonary agenesis by the existence of the rudimentary blind-ending bronchus which can be depicted in detail on CT (Fig. 2).

Not only agenesis of a lung, but also agenesis of a lobe may occur and simulate lobectomy or collapse of a lobe [6].

**Fig. 3:**
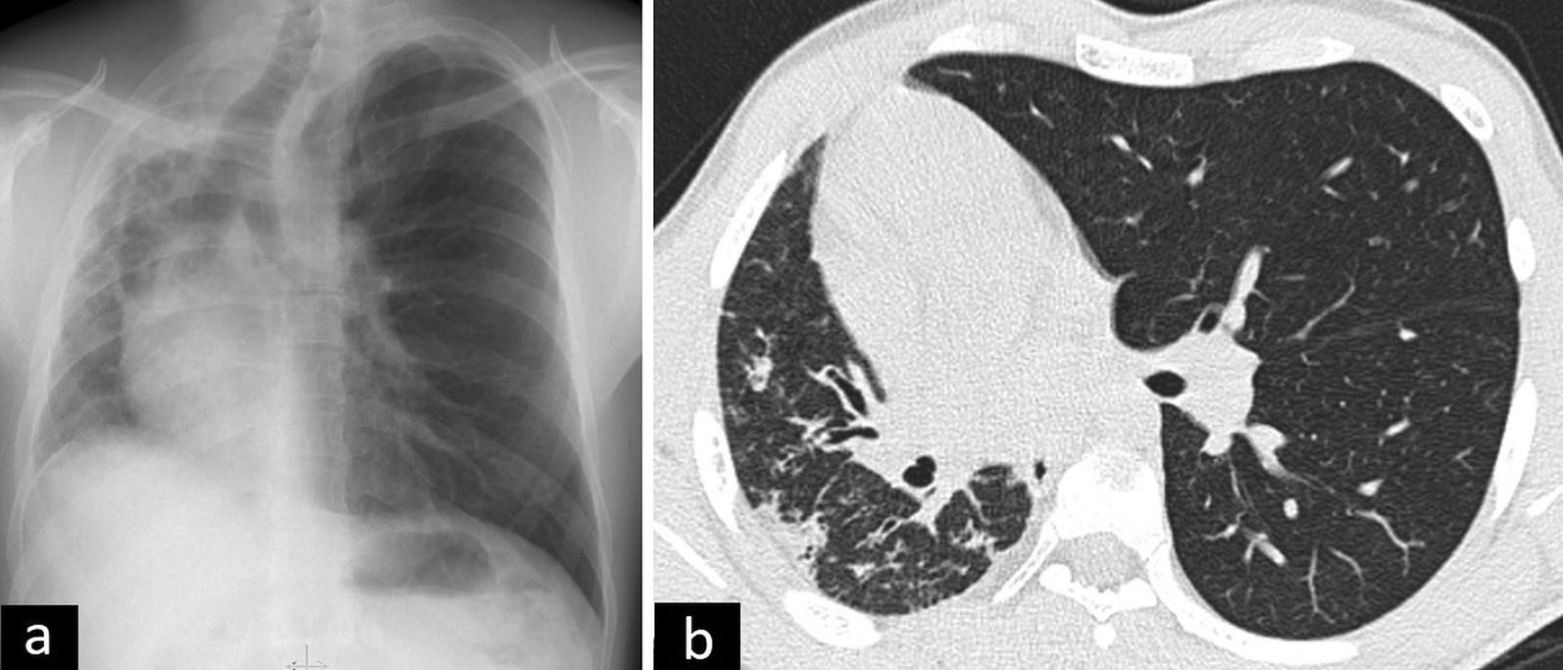
Imaging findings of pulmonary hypoplasia. Chest X-ray (**a**) and axial CT image (**b**) of a 26-year-old male presenting with recurrent infection demonstrate pulmonary hypoplasia of the right lung. Although bronchi and alveoli are present in underdeveloped lungs, the size of the right lung is decreased. Consolidations due to infection can also be seen on axial CT (**b**)

Imaging features of pulmonary hypoplasia are a decrease in ipsilateral lung volume and pulmonary artery, narrowing of intercostal spaces, compensatory hyperinflation, and mediastinal shift [7] (Fig. 3).

Underdevelopment anomalies in adults can be asymptomatic or symptomatic as a result of recurrent infections or pulmonary hypertension. Recurrent infections can also cause bronchiectasis which may lead to recurrent hemoptysis [4, 5, 8].

### Congenital pulmonary airway malformation (CPAM)

CPAM was previously called congenital cystic adenomatoid malformation, but due to the absence of adenomatoid and cystic changes in all types of the disease, CPAM is now accepted as a more appropriate name for these congenital lung diseases. CPAM is divided into 5 types (0–4) according to the recent classification. Type 0, a very rare and lethal type of CPAM, consists of severe acinar dysgenesis or airway dysplasia. Type 1, the most common type and most frequently seen in adults because of its better prognosis, presents with one or multiple large cysts (2–10 cm). Type 2, probably lethal because of the associated cardiac and renal anomalies, is defined by solitary or multiple cysts 0.5–2 cm in size. Type 3, which has a poor prognosis and is rarely seen in adults, involves the entire lobe and appears solid due to microcysts (< 0.5 cm). Type 4 typically affects a single lobe and can be confused with pleuropulmonary blastoma because of large cystic lesions on radiological images [3, 9–11].

**Fig. 4:**
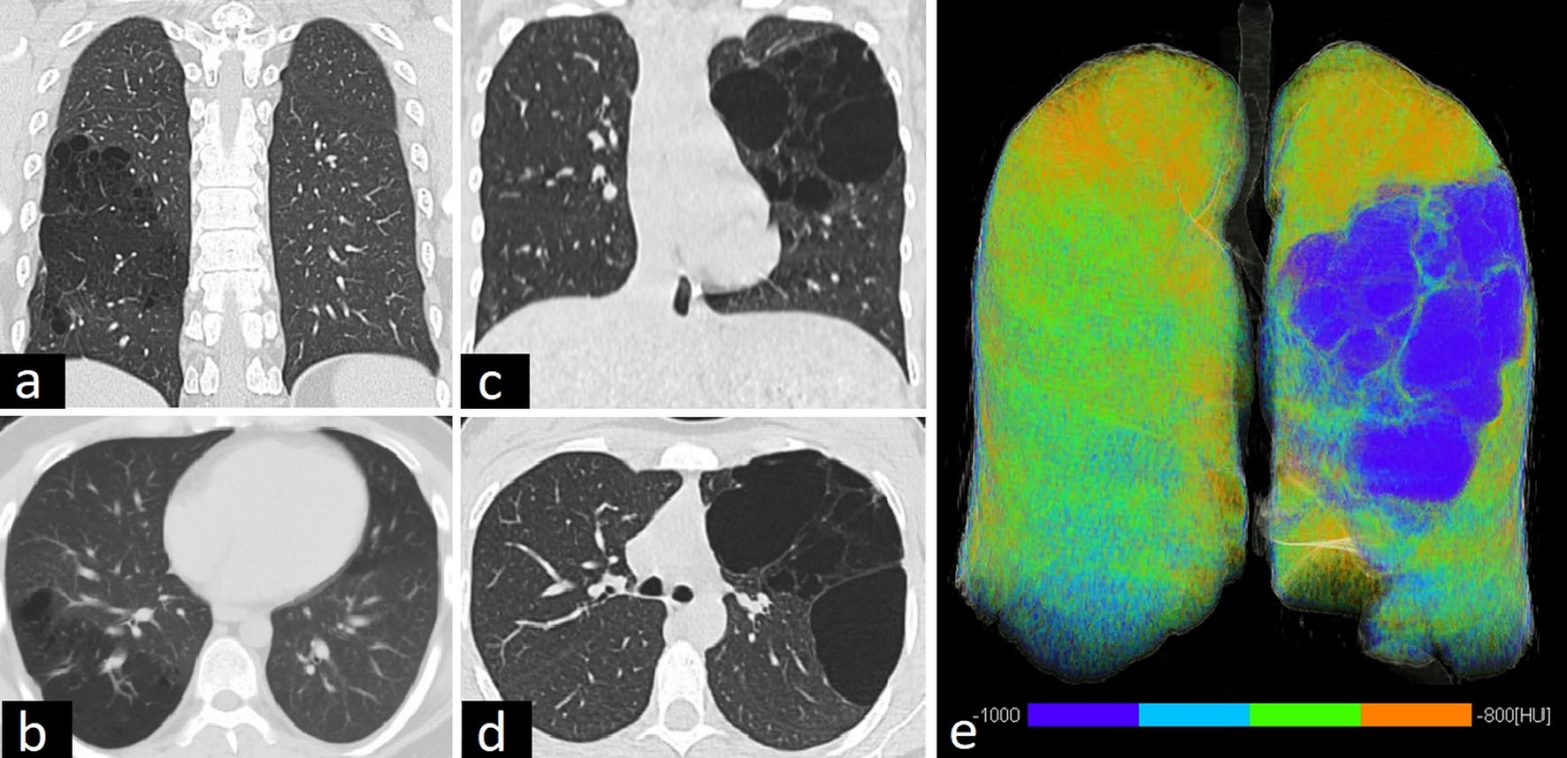
CT findings of congenital pulmonary airway malformation (CPAM) in two adult patients. Cysts < 2 cm in type 2 CPAM (**a**, **b**) and > 2 cm in type 1 CPAM (**c**, **d**) are seen on coronal and axial CT images. Quantitative 3D reconstruction image (**e**) depicts hyperinflation and lower lung density in the patient with type CPAM 1 (blue area)

The radiological features of CPAM vary according to the type and the presence of superinfection. One or multiple cysts, filled with air or air-fluid levels, are seen in type 1 CPAM. Type 2 manifests as air-filled multicystic masses or consolidation. Type 3 presents as a solid mass because of microcysts [1–3] (Fig. 4).

Both other congenital diseases such as sequestration and non-congenital diseases such as infectious diseases and malignancies should be considered in the differential diagnoses of CPAM. However, all of these diseases can accompany CPAM. Malignant transformation of CPAM may be seen as adenocarcinoma, rhabdomyosarcoma, and pleuroblastoma [12, 13]. Systematic supply which is characteristic of sequestration can help distinguish sequestration from CPAM, whereas the coexistence of intralobar sequestration and CPAM can be seen in hybrid lesions. Infectious diseases such as Staphylococcus aureus, Klebsiella, and Streptococcus pneumonia can cause pneumatoceles which are characterized by gas-filled, thin-walled cystic spaces and mimic CPAM [14]. As CPAM is located in the lower lobes, it may also be misdiagnosed as a diaphragmatic hernia. Because herniated bowel loops can mimic multiple cystic structures. Coronal and sagittal reformatted images can help to distinguish CPAM from a diaphragmatic hernia.

CPAM in adults can be asymptomatic or symptomatic as a result of recurrent infections. An enhanced, thickened cyst wall can be seen in infected CPAM. Complete surgical resection is recommended because of possible malignant transformation [4, 12, 13, 15].

### Congenital lobar hyperinflation (CLH)

CLH, formerly known as congenital lobar emphysema, is hyperinflation of one or multiple lobes. The left upper and right middle lobes are most commonly affected, while the lower lobes are rarely affected. Although CLH is usually diagnosed in the neonatal period, it may usually be seen in adults. CLH is more common in males [1, 16].

**Fig. 5:**
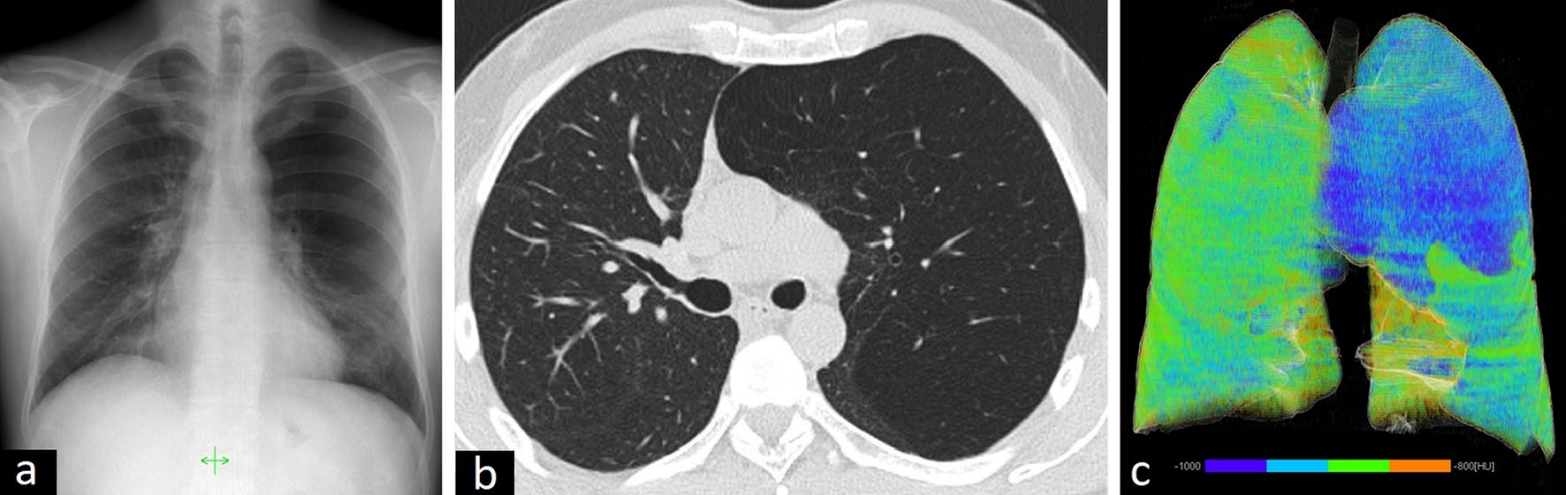
Radiological features of congenital lobar hyperinflation in a 42-year-old male presenting with frequent respiratory infections. Chest X-ray shows hyperlucency of the left lung (**a**). Axial CT image (**b**) and quantitative 3D reconstruction image (**c**) demonstrate lobar hyperinflation (**c**, blue area) and shift to the contralateral side

The radiological features of CLH are localized hyperlucency of the affected lung, attenuated and displaced vessels, and mass effect on adjacent structures including compressive atelectasis, mediastinal shift to the contralateral hemithorax, separated ribs, and flattened diaphragm on the ipsilateral side [1, 10, 16] (Fig. 5).

The radiological appearance of hyperlucency of the affected lung can be the result of a decreased pulmonary vascularity or a hyperinflation process. So, various congenital and acquired disorders can mimic CLH. Underdevelopment anomalies of the lung and agenesis of pulmonary vasculature can mimic CLH because of the hyperinflation of the contralateral lung on radiography. But they can easily be differentiated on CT images by demonstrating the bronchopulmonary and vascular structures. Also, congenital disorders including bronchial atresia and CPAM can be listed as the differential diagnosis of CLH. Bronchial atresia, which can also coexist with CLH, is considered in the differential diagnosis of CLH. Although both CLH and bronchial atresia show hyperinflation, mucoceles in patients with bronchial atresia aid in distinguishing it from CLH. Furthermore, pulmonary hyperinflation which is caused by the obstruction of the airway due to a foreign body or mass can be confused with CLH on chest X-ray [16].

Adults with CLH can present with frequent respiratory infections, chest pain, and shortness of breath, or it may be diagnosed incidentally. Symptomatic CLH requires surgical resection and has an excellent prognosis [3, 16, 17].

### Rare hereditary diseases that can manifest with lung cysts

#### Birt–Hogg–Dubé Syndrome (BHDS)

BHDS is an infrequent autosomal dominant multisystemic disease occurring due to a mutation in the folliculin gene which is located on chromosome 17. BHDS is characterized by cutaneous fibrofolliculomas, pulmonary cysts, and kidney tumors of varied histological types. Although BHDS is usually seen in the 3rd or 4th decade of life, it can be seen in individuals at any age without certain gender predilection [18, 19].

**Fig. 6:**
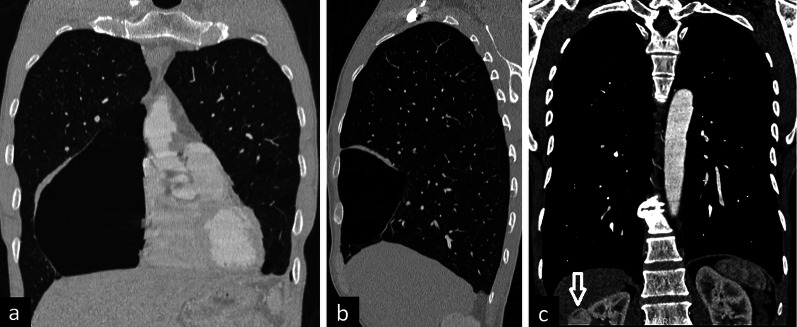
A 53-year-old man with Birt–Hogg–Dubé Syndrome. Coronal (**a**) and sagittal (**b**) reconstruction images in the lung window show large cysts with irregular shapes. Contrast-enhanced coronal reconstruction image in the mediastinal window (**c**) demonstrates an enhancing heterogeneous solid mass in the right kidney

The characteristic chest CT findings of BHDS are multiple, thin-walled cysts with medial and lower zone predominance. The cysts can be varied in size and shape. Large cysts are usually irregular in shape and multiseptated. Subpleural and fissural cysts can also be usually seen. Spontaneous pneumothorax because of rupture of the cysts can be detected on CT images. Besides lung findings, renal masses can also be incidentally seen on CT scans (Fig. 6).

Differential diagnosis of BHDS includes other diffuse cystic lung diseases such as lymphangioleiomyomatosis, pulmonary Langerhans cell histiocytosis, and neurofibromatosis type 1. The morphology, size, and distribution of the lung cysts can be helpful in the differentiation of BHDS from other cystic lung diseases.

The management of BHDS primarily depends on the early diagnosis of disease. Identification of affected family members, early diagnosis and treatment of kidney masses, and avoiding pneumothorax are vital in disease management. Pleurodesis can be performed for preventing recurrent pneumothorax [19].

#### Lymphangioleiomyomatosis (LAM)

LAM is a rare multisystemic disease that can occur sporadically or with tuberous sclerosis complex (TSC). TSC is an autosomal dominant disease that usually affects women over 30 years of age. It is characterized by benign tumors affecting the brain, skin, heart, kidneys, and lungs [20, 21].

**Fig. 7:**
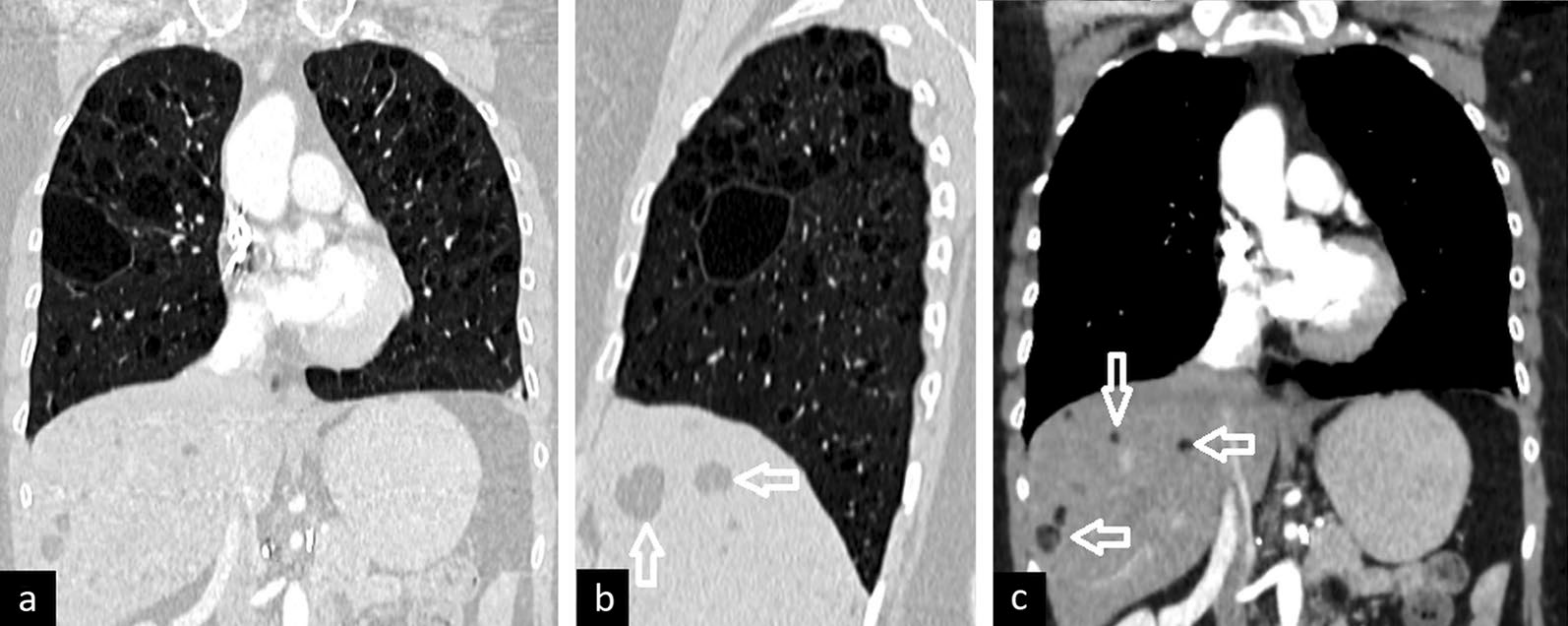
A 32-year-old woman with lymphangioleiomyomatosis. Bilateral multiple thin-walled cysts without zonal distribution are seen on contrast-enhanced coronal (**a**) and sagittal (**b**) reconstruction images in the lung window. Coronal image in the mediastinal window depicts fat containing liver lesions compatible with angiomyolipomas (arrows)

On chest CT, thin-walled, round, air-filled numerous cysts are distributed throughout both lungs without zonal predominance. Spontaneous pneumothorax is a common finding. Also, pleural effusion especially in chylous nature can occur. Furthermore, extrathoracic manifestations including benign renal and liver tumors called angiomyolipomas may be detected on chest CT and facilitate the diagnosis of LAM (Fig. 7).

Multiple lung cysts without zonal distribution can help distinguish LAM from the other diffuse cystic lung diseases. The cysts in LAM are smaller, more uniform in shape, and more numerous than the cysts in BHDS.

Patients with LAM should avoid smoking and be vaccinated against common respiratory pathogens similar to individuals with other chronic lung diseases. Supplemental oxygen for hypoxemia, pulmonary rehabilitation, and bronchodilators can be performed according to the clinical status of the patients. Conservative management is usually preferred for the complications such as pneumothorax and chylous effusions. Sirolimus as an immunosuppressant can be given to patients with LAM and abnormal lung function. Lung transplantation can be performed for patients with severe disease [20].

#### Neurofibromatosis type 1 (NF 1)

NF 1 is an autosomal dominant multisystemic disease that is characterized by café au lait spots, axillary and inguinal freckling, Lisch nodules, neurofibromas, and tumors throughout the nervous system.

**Fig. 8:**
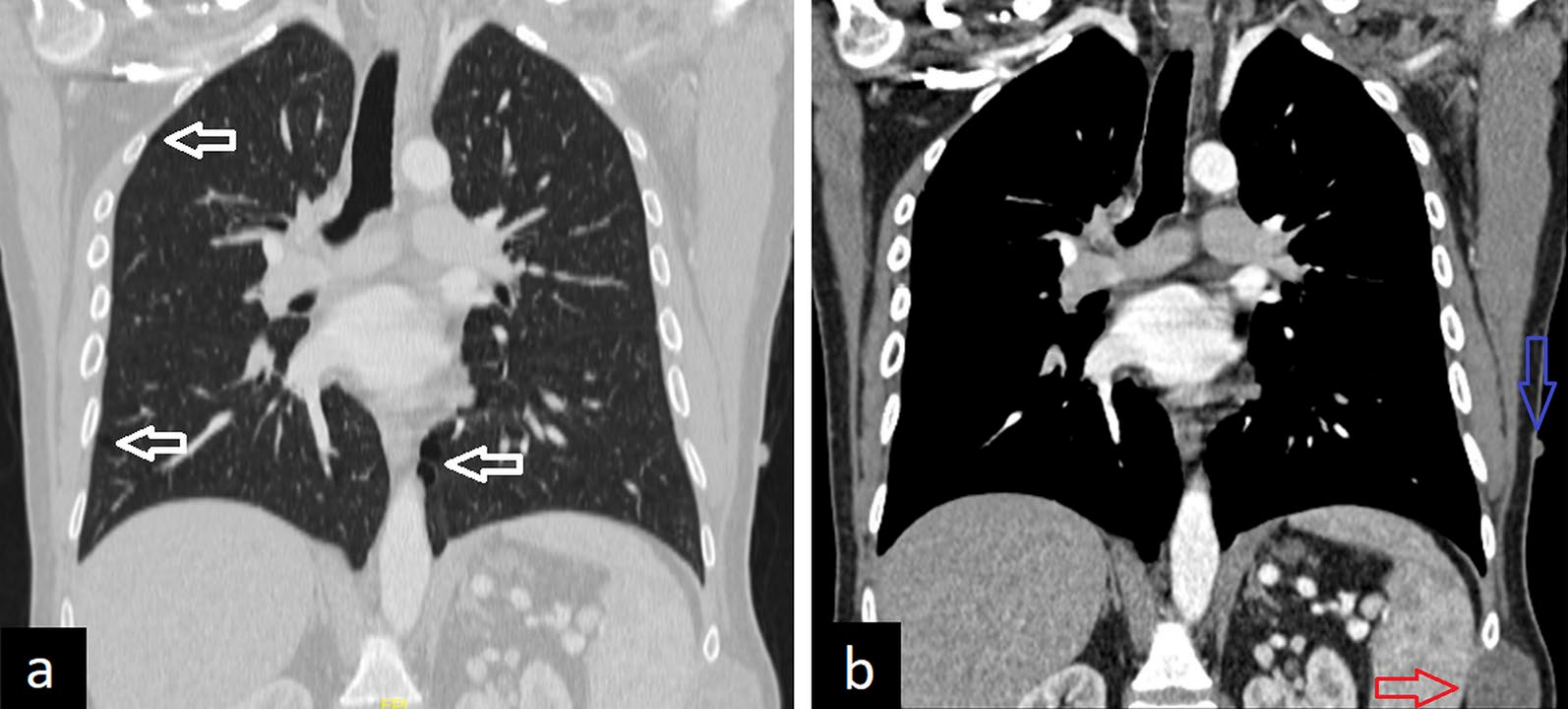
A 32-year-old man with neurofibromatosis type 1. Coronal (**a**) reconstruction CT image in the lung window shows lung cysts (white arrows). Cutaneous neurofibroma (blue arrow) and neurofibroma arising from the intercostal nerve (red arrow) are seen on the coronal reconstruction image in the mediastinal window (**b**)

Lung parenchymal involvement is a rare manifestation of NF 1 and includes cysts and bullae, lower lobe predominant interstitial fibrosis, nodules, and ground-glass opacities. The incidence of interstitial lung disease in NF 1 is approximately 6–12%, while the incidence of cysts in NF 1 is about 15–25% [22–24]. Cysts are thin-walled and usually show peripheral and upper lung distribution. However, they can also be located in the central and lower lung areas. The number and sizes of the cysts are various. Besides lung parenchymal findings of NF 1, mediastinal, intercostal, and cutaneous neurofibromas, scoliosis, and lateral meningoceles can be observed on thorax imaging (Fig. 8).

### Bronchogenic cyst

Bronchogenic cysts are components of the foregut duplication cysts that are classified as bronchogenic, enteric, and neurenteric cysts. A certain diagnosis of bronchogenic cysts can be performed if respiratory epithelium is seen in pathological examination [25, 26]. The bronchogenic cysts are generally located in the middle mediastinum (65%–90%), especially the subcarinal region, but they can also take place in the lung parenchyma (12%–20%) or unusual extrathoracic locations [1, 2, 26, 27].

**Fig. 9:**
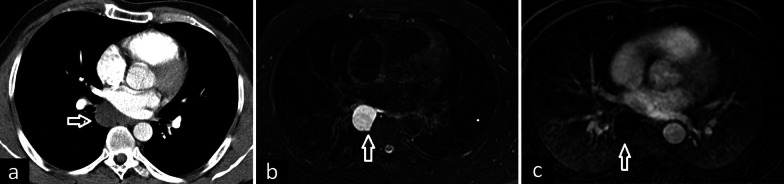
CT and MRI features of bronchogenic cyst. Low-density mass behind the left atrium is seen on contrast-enhanced axial CT image (**a**, arrow). Axial T2-weighted MR sequence (**b**) demonstrates uniform high signal intensity similar to cerebrospinal fluid and contrast-enhanced T1-weighted image (**c**) demonstrates the absence of contrast enhancement within the cyst (arrows)

**Fig. 10:**
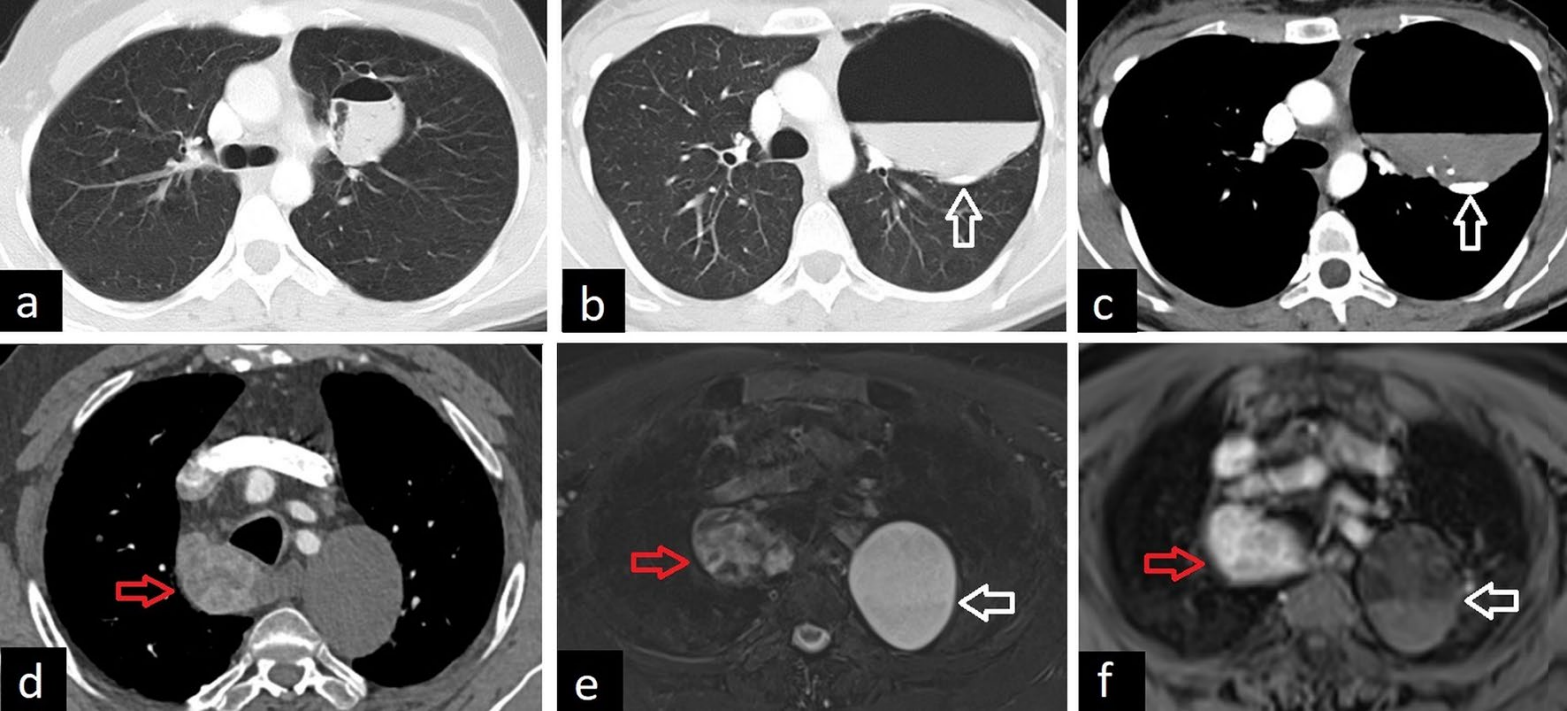
Bronchogenic cysts showing air-fluid and fluid–fluid levels. Axial CT image in lung window in a patient shows air-fluid level within the bronchogenic cyst (**a**). In another patient, air-fluid level and also milk of calcium can be seen within the bronchogenic cyst on axial CT images in lung (**b**, arrow) and mediastinal windows (**c**, arrow). Axial CT image in mediastinal window in another patient shows a bronchogenic cyst (**d**, white arrow). Fat suppressed T2 weighted (**e**, white arrow) and post-contrast T1 weighted (**f**, white arrow) MR images demonstrate the fluid-fluid level within the cyst. The red arrows show the ectopic thyroid nodule (**d**–**f**)

The characteristic radiological feature of bronchogenic cysts is a well-defined, round, or oval solitary lesion. Peripheral calcifications or septations can occur. A thin wall with a slight contrast enhancement or without contrast enhancement is usually detected on contrast-enhanced CT images [2, 28]. Cyst attenuation can vary depending on the amount of internal proteinaceous substances. Soft tissue density owing to proteinaceous contents on CT can be confused with lymphadenopathy or a solid mass. MRI can provide a more reliable diagnosis for cysts with proteinaceous content. Simple cysts show signal intensity like cerebrospinal fluid, with hypointensity on T1, and hyperintensity on T2-weighted sequences, while the intensity of proteinaceous cysts appears similar to the skeletal muscle. No enhancement in the central part of the lesion after gadolinium administration can aid in distinguishing proteinaceous bronchogenic cysts from lymphadenopathy or a solid mass (Figs. 9, 10).

**Fig. 11:**
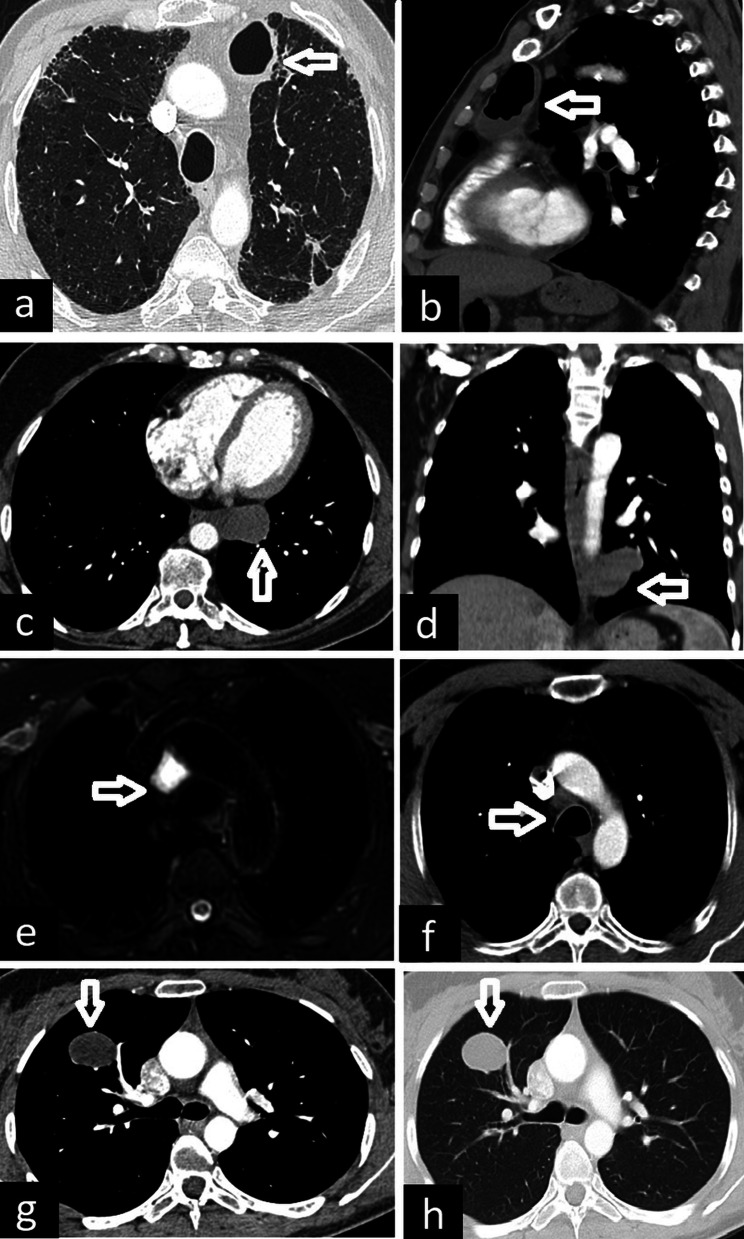
Differential diagnoses of bronchogenic cyst. Infected bullae on axial (**a**) and sagittal (**b**) CT images are observed. Esophageal duplication cyst on axial (**c**) and coronal (**d**) CT images, pericardial cyst on T2-weighted MRI (**e**) and axial CT image (**f**) and hydatid cyst on axial CT images (**g**, **h**) are considered in the differential diagnoses of bronchogenic cyst

Differential diagnoses of bronchogenic cysts include other congenital cysts such as pericardial cysts and esophageal duplication cysts. Esophageal duplication cyst and bronchogenic cyst cannot be differentiated radiologically and require histopathological assessment. However, more tubular appearance, close relationship with the esophagus, and a relatively thick wall on CT and MRI images favor esophageal duplication cyst. In addition to the congenital reasons, non-congenital cystic lesions such as hydatid cysts, infected bullae, necrotic lymphadenopathy, and masses can be listed as the differential diagnoses of the bronchogenic cysts [3, 4, 29, 30] (Fig. 11).

Adults with bronchogenic cysts can be asymptomatic especially when the cyst is small or may present with symptoms such as cough, pain, dysphagia, and difficulty in breathing due to compression of the esophagus and airways. Hyperinflation or collapse because of the effect of the mass can be demonstrated on CT images. Radiological findings such as air-fluid level and thickened enhanced cyst wall can also be helpful in the diagnosis of infected bronchogenic cysts, which may mimic an infected bulla, lung abscess, or ruptured hydatid cysts.

Percutaneous, transbronchial, and transesophageal aspiration can be performed in treatment, but the cyst may eventually re-occur. Although bleomycin sclerosis with aspiration reduces the risk of recurrence, complete surgical resection provides definitive treatment [31, 32].

### Abnormal bronchi

Congenital abnormal bronchial diseases can be listed as tracheal bronchus, cardiac bronchus, bronchial atresia, and bronchial dilatation.

#### Tracheal bronchus

**Fig. 12:**
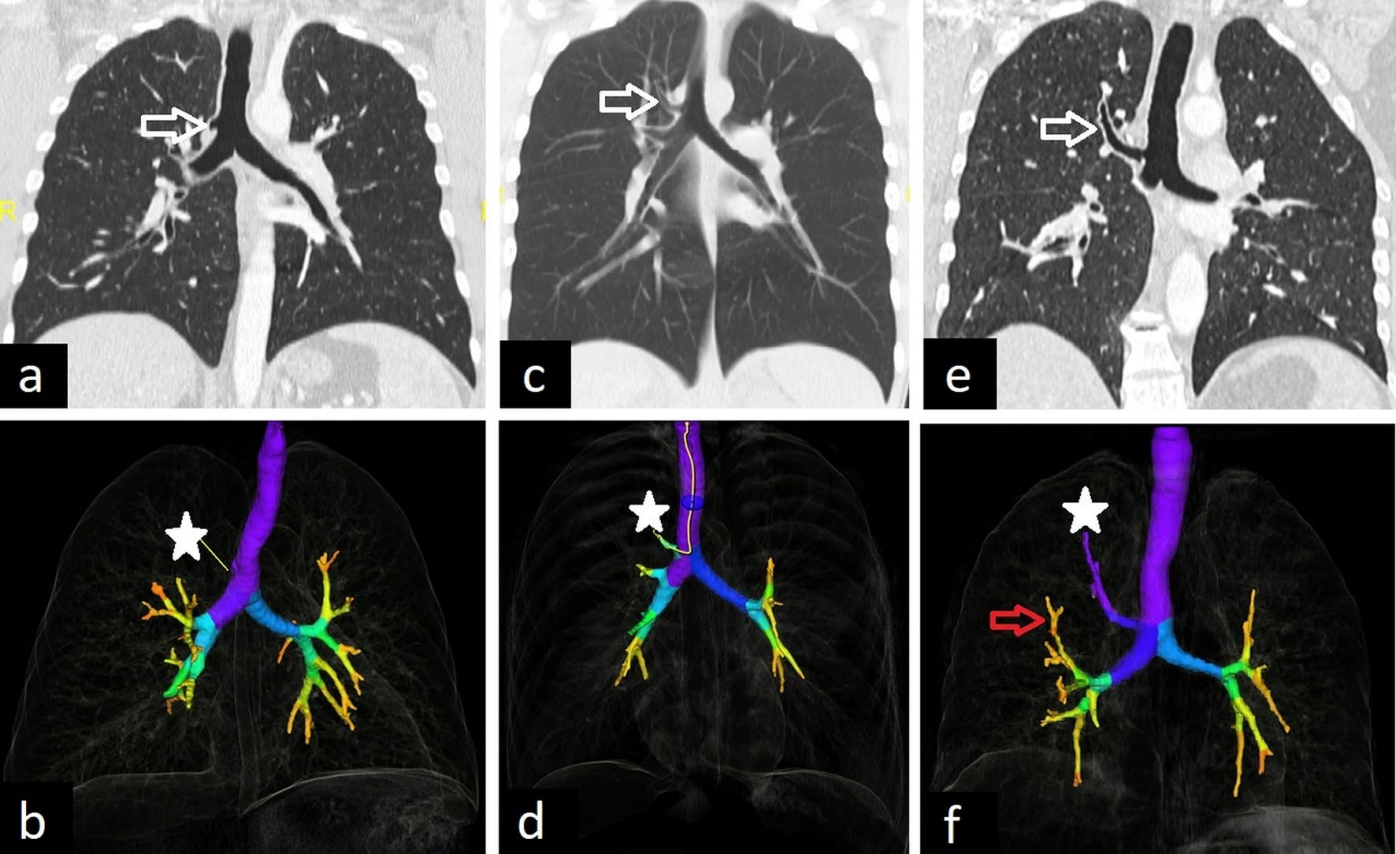
CT images of tracheal bronchi in different patients. Blind-ended tracheal bronchus is seen on coronal (**a**, arrow) and 3D reconstruction (**b**, star) CT images. Tracheal bronchi with accompanying lung tissue are demonstrated on coronal (**c**, **e**, arrow) and 3D reconstruction (**d**, **f**, star) CT images in two patients. The tracheal bronchus supplies the apical and posterior segments of the right upper lobe (**e**, arrow) and the right middle bronchus supplies the anterior segment of the right upper lobe (**f**, red arrow) in a 43-year-old asymptomatic male patient

The tracheal bronchus (also known as pig bronchus) originates from the right wall of the trachea, usually within 2 cm of the carina. There are two types: supernumerary, in which the right upper lobe bronchus trifurcates normally, and displaced, in which the right upper lobe bronchus bifurcates and the displaced bronchus supplies the apical segment [1]. It may also end blindly. Besides axial CT images, coronal reconstruction and 3D reconstruction images can depict abnormal bronchus (Fig. 12).

Although tracheal bronchus is usually incidentally detected, recurrent pneumonia and bronchiectasis can be observed in adults [33].

#### Cardiac bronchus

**Fig. 13:**
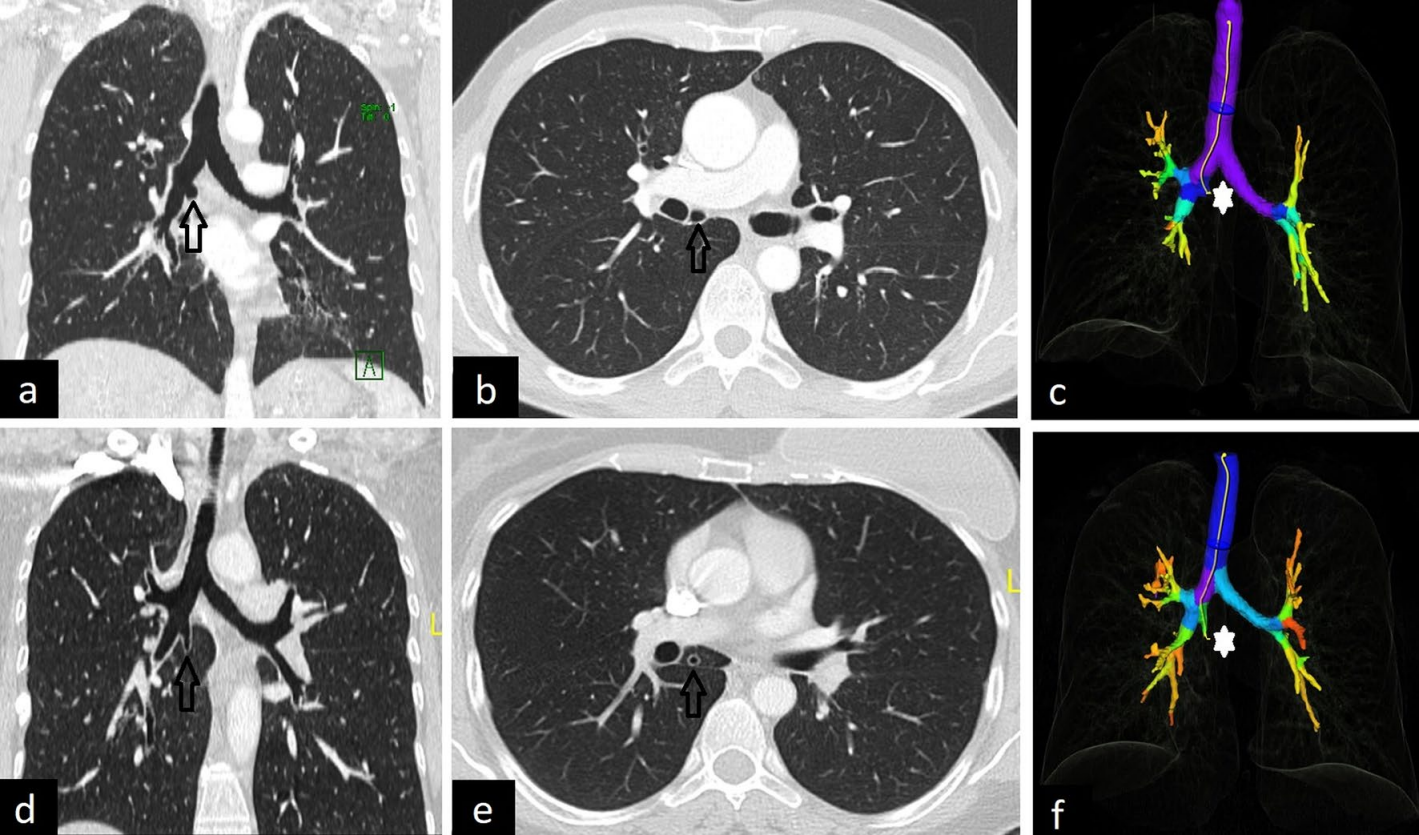
CT images of cardiac bronchi in two patients. Blind-ended cardiac bronchus is seen on coronal (**a**, arrow), axial (**b**, arrow) and 3D reconstruction (**c**, star) CT images. In another patient coronal (**d**, arrow), axial (**e**, arrow), and 3D reconstruction (**f**, star) CT images demonstrate cardiac bronchus with accompanying lung tissue and pleural fissure

The cardiac bronchus originates from the inferomedial of the right main or intermediate bronchus. It may end blindly or be accompanied by lung tissue. In some cases, a clear pleural fissure, bounding the lung tissue, can also be seen on CT images (Fig. 13).

Although the majority of cases of cardiac bronchus in adults are discovered incidentally, it can cause repeated infection and hemoptysis [34, 35].

#### Tracheal/bronchial diverticula

Diverticula of the trachea and bronchus can be congenital and acquired. The congenital diverticula are usually located in the posteromedial border of the right main bronchus or posterolateral border of the right trachea. Congenital diverticula contain the respiratory epithelium, smooth muscle, and cartilage, while acquired diverticula only affect respiratory epithelium. Acquired tracheal diverticula may occur due to the increased intraluminal pressure and weakened tracheal wall resulting from chronic cough, bronchitis, bronchiectasis, emphysema, and respiratory infections [21, 36].

**Fig. 14:**
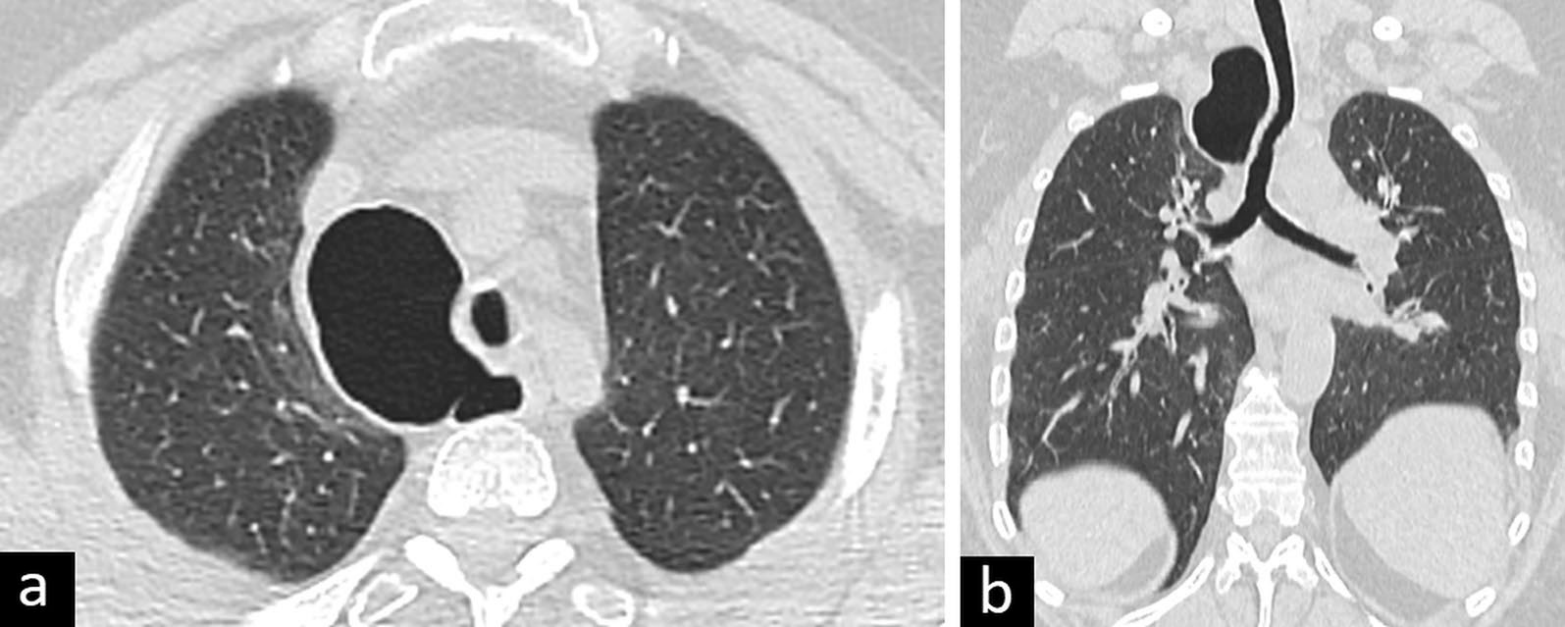
Tracheal diverticula in a 61-year-old woman presenting with dyspnea. Axial (**a**) and coronal (**b**) CT images demonstrate a giant right-sided paratracheal cyst

CT can show both paratracheal cyst and communication to the tracheal lumen. But, the connection between the diverticula and airway is not always seen by radiological methods. Differential diagnoses of tracheal/bronchial diverticula are pharyngocele, Zenker’s diverticula, and lung bullae [37, 38] (Fig. 14).

They are usually asymptomatic and incidentally detected. Cough, sputum, dyspnea, dysphagia, and tracheobronchitis may be observed in symptomatic patients. Surgical resection may be performed in young and symptomatic patients. Conservative treatment including antibiotics, mucolytic drugs, and physiotherapy can be preferred in older patients [21, 36].

#### Bronchial atresia

Congenital bronchial atresia is characterized by focal obstruction of a subsegmental or segmental bronchus with a normal distal airway. The most commonly affected lobe is the left upper lobe. It is followed by the right upper, middle, and lower lobes [3, 10].

**Fig. 15:**
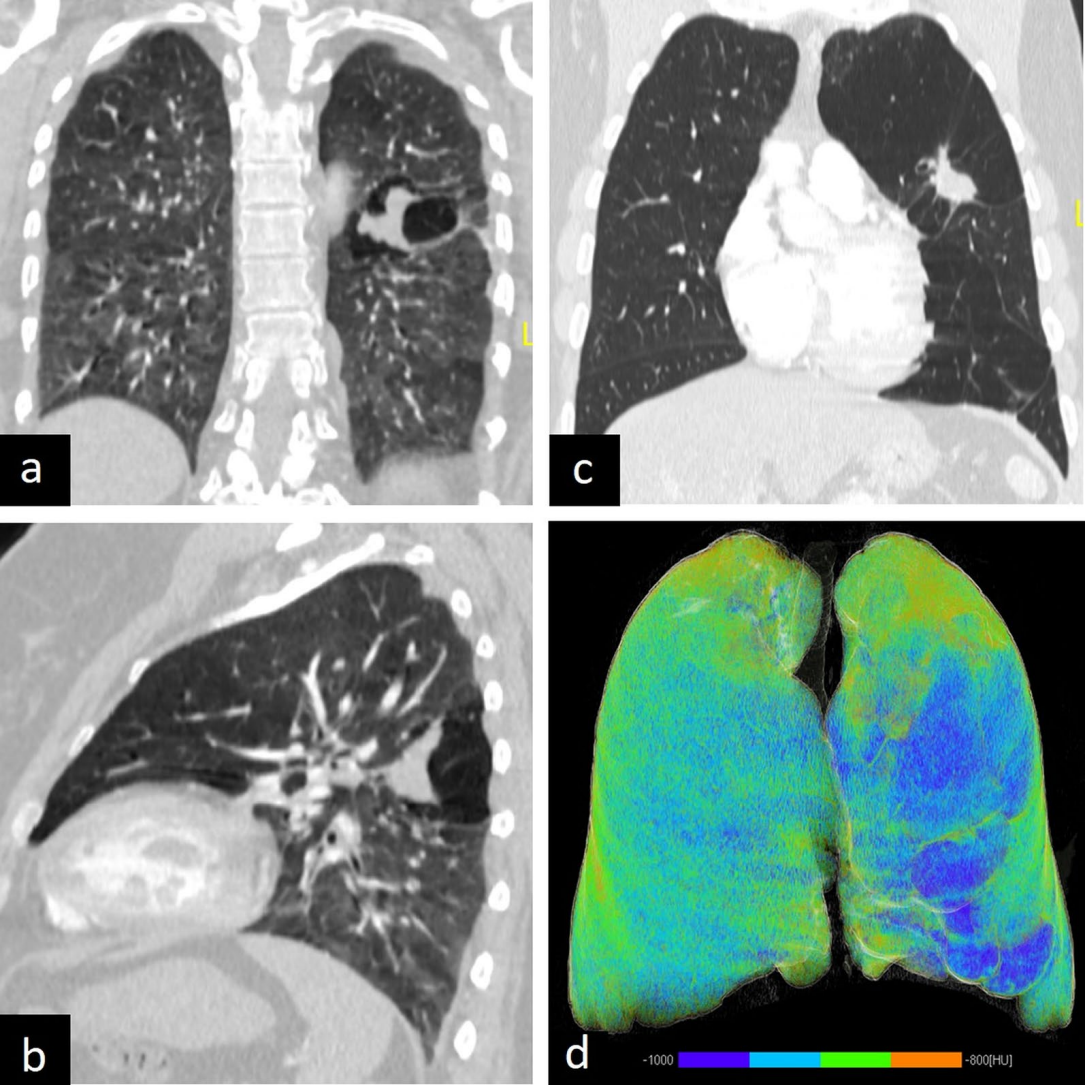
Imaging features of bronchial atresia in two asymptomatic patients. Central mass-like opacity because of the mucus-filled bronchus is seen on coronal (**a**, **c**) and sagittal (**b**) CT images. Coronal (**a**) and sagittal (**b**) CT images in the expiratory phase and quantitative 3D reconstruction (**d**) image clearly show hyperinflation (**d**, blue area) due to air trapping

The characteristic radiological finding of bronchial atresia is a central mass-like opacity corresponding to a mucus-filled bronchus distal to the region of the atresia. Opacity may show tubular configuration and finger-in-glove appearance or a rounded shape because of mucoid impaction with or without air-fluid level. Another distinctive feature of bronchial atresia is hyperinflation of the obstructed segment. Hyperinflation can occur because of both collateral air drift and decreased vascular supply in the involved segment of the area. Hyperinflation can also be depicted with quantitative 3D reconstruction images created according to lung density (HU) values [10, 39] (Fig. 15).

**Fig. 16:**
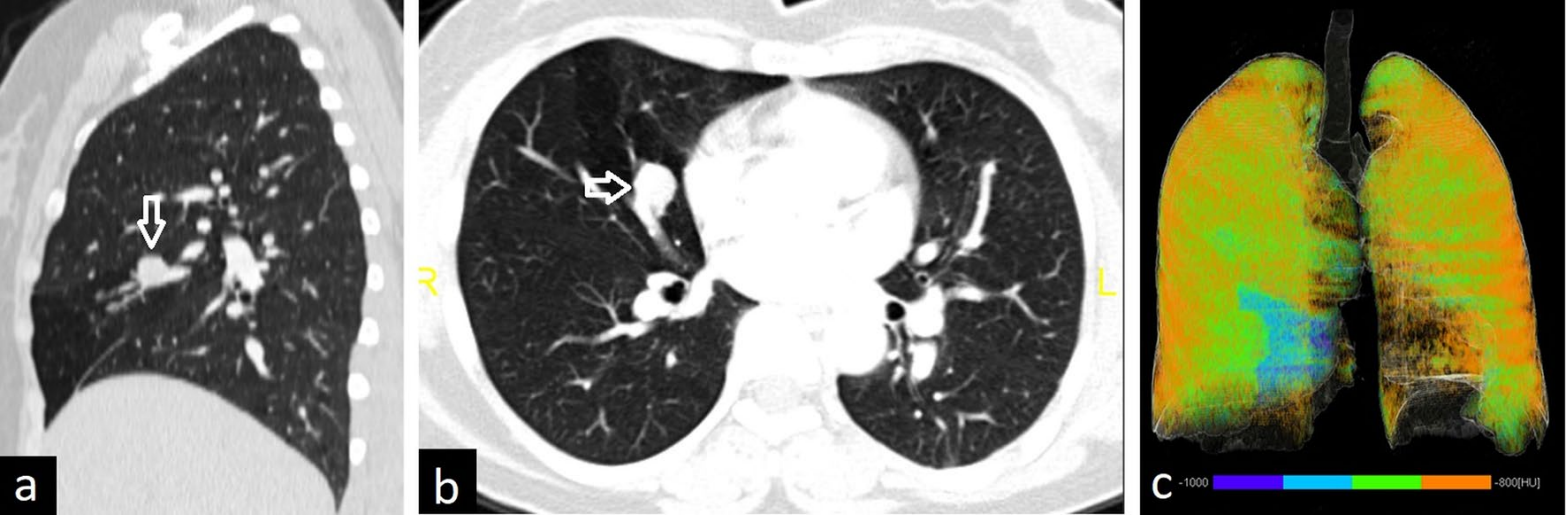
Bronchial carcinoid mimicking bronchial atresia. Sagittal (**a**), axial (**b**), and quantitative 3D (**c**) CT images show that a bronchial carcinoid (arrows) can mimic bronchial atresia because of air-trapping in the distal segment of the mass

Bronchial atresia can be confused with CLH because of hyperinflation. The absence of mucus-filled dilated bronchus in CLH can help in differentiation. In addition, bronchial carcinoids should be considered in the differential diagnosis of bronchial atresia in adults, as bronchial carcinoids may also cause air trapping and mucoid impaction similar to bronchial atresia. Contrast-enhanced CT or MRI can help distinguish bronchial atresia from carcinoids as the latter may show central enhancement within the mucoid impaction. Moreover, the signal intensity of carcinoids may not be as high as that of mucoceles on T2-weighted MRI [4] (Fig. 16).

Although most patients with bronchial atresia are diagnosed incidentally, some may present with recurrent pneumonia and elective surgical excision may be performed.

#### Bronchial dilatation

Bronchial dilatation due to congenital lung diseases in adults can be listed as Mounier-Kuhn, primary ciliary dyskinesia, Williams–Campbell, and cystic fibrosis. These diseases can imitate each other and other acquired diseases which present with bronchiectasis. Allergic bronchopulmonary aspergillosis, chronic aspiration and recurrent infectious diseases, severe obstructive lung diseases, and traction bronchiectasis because of interstitial lung diseases are non-congenital diseases that can mimic congenital diseases presenting with bronchial dilatation. Accompanying clinical findings and some imaging findings such as involvement of the upper or lower zones of the lung, the involvement of the main airways, or the type of bronchiectasis can be helpful in the differential diagnosis.

**Fig. 17:**
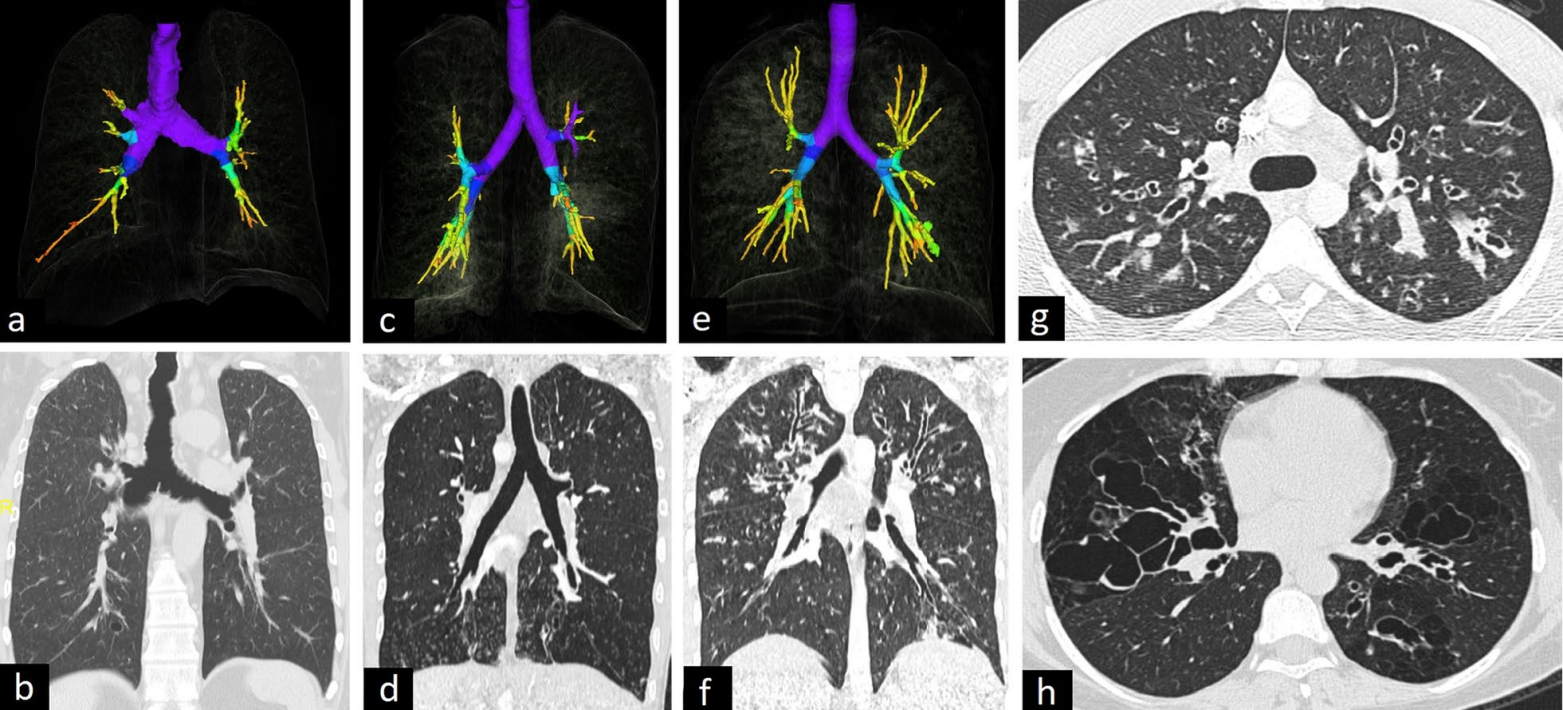
Imaging findings of congenital diseases causing bronchial dilatation. 3D reconstruction (**a**) and coronal CT image (**b**) show dilatation of the main airways in a patient with Mounier-Kuhn syndrome. Bronchiectasis, centrilobular opacities, and increased density predominantly affecting the lower lobes of the lungs are seen in a patient diagnosed with Kartagener (**c, d**). Upper lobe-predominant bronchiectasis and thickened bronchial walls are observed in cystic fibrosis (**e**–**g**). Severe and bilateral cystic bronchiectasis in the subsegmental bronchi (4th and 6th generation) is seen in a patient with Williams–Campbell syndrome (**h**)

Mounier-Kuhn syndrome, also known as tracheobronchomegaly, is a rare disease that is most commonly seen in middle-aged men. Lack of smooth muscle and elastic fibers within the wall of the trachea and main bronchi causes dilatation and laxity of the airways, causing a marked enlargement to be seen in the inspiration phase, and airway collapse may occur in the expiration phase on CT images. The transverse diameters of the main airways are measured larger than 3 cm for the trachea, and 2.4 cm, and 2.3 cm for the main right and left bronchi, respectively. Chronic productive cough and recurrent pulmonary infections can be seen in adults and conservative treatment such as physiotherapy and postural drainage can be applied [40, 41] (Fig. 17).

Primary ciliary dyskinesia, an autosomal recessive disease, is a result of a congenital defect in ciliary structure and function. Kartagener’s syndrome is a subtype of primary ciliary dyskinesia and is defined by the clinical triad of bronchiectasis, situs inversus, and chronic sinusitis. The main imaging findings are bronchial wall thickening and bronchiectasis, predominantly affecting the middle and lower lobes of the lung. Chronic pulmonary infection especially P. aeruginosa, hemoptysis, and eventually respiratory failure can be seen as complications of primary ciliary dyskinesia. Physiotherapy, treatment of chest infection, and immunization can be performed for the treatment [42, 43] (Fig. 17).

Williams–Campbell syndrome results from cartilage abnormality in the 4th and 6th order subsegmental bronchi. The typical radiological finding is severe, bilateral, and symmetrical cystic bronchiectasis in the subsegmental bronchi usually accompanied by bronchial wall thickening, mucus impaction, and bronchomalacia. The trachea and main bronchi are preserved. In dynamic images, the abnormal bronchi show ballooning in the inspiration phase and collapse in the expiratory phase [44, 45] (Fig. 17).

Cystic fibrosis is an autosomal-recessive disorder causing defective mucociliary clearance and primarily affecting the lungs. The chest CT findings of cystic fibrosis can be listed as follows: upper lobe-predominant bronchiectasis, thickened bronchial walls, mucoid impaction, mosaic attenuation, tree-in-bud, lymphadenopathy, pulmonary artery and bronchial artery dilatation. Despite the advances in treatment, respiratory failure and pulmonary complications account for 95% of morbidity and mortality [46, 47] (Fig. 17).

## Vascular anomalies

Vascular anomalies can be listed as anomalies of the pulmonary artery, vein, bronchial artery, and pulmonary arteriovenous malformation.

### Anomalies of the pulmonary artery

#### Proximal interruption of the pulmonary artery (PIPA)

PIPA, also known as the unilateral absence of pulmonary artery, results from when the proximal part of the main pulmonary artery fails to appear in embryological development. It can occur on both sides, although it is more commonly located on the right side. Left-sided anomaly is generally related to other congenital cardiovascular diseases [48].

**Fig. 18:**
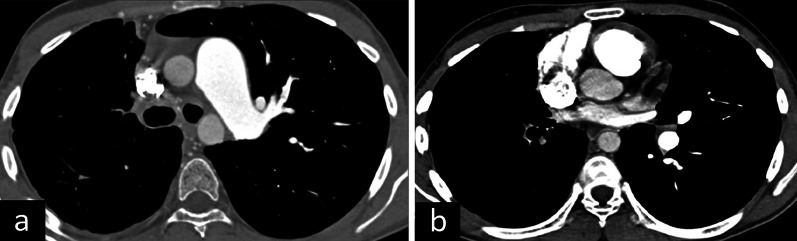
A 20-year-old man with proximal interruption of the pulmonary artery (PIPA). Contrast-enhanced axial CT images (**a**, **b**) show the lack of the proximal portion of the right main pulmonary artery

Characteristic imaging features are the lack of the proximal pulmonary artery within 1 cm from the main pulmonary artery origin, hypoplasia of the involved side, hyperinflation of the contralateral lung, and mediastinal shift to the ipsilateral side (Fig. 18). Although the bronchial anatomy is normal, the affected lung is hypoplastic and lucent due to a relative decrease in vascular structures. Contrast-enhanced CT images can demonstrate dilated contralateral pulmonary artery and supply of the distal pulmonary artery branches from the aortopulmonary collateral arteries, including bronchial, internal mammary, intercostal, subdiaphragmatic, or coronary arteries. Moreover, the affected lung parenchyma can show mosaic attenuation, fibrotic changes, bronchiectasis, intraparenchymal and subpleural cysts [3, 10, 48].

PIPA can be confused with pulmonary hypoplasia. However, demonstration of vascular structures on CT or MR angiography helps in the differentiation of PIPA from pulmonary agenesis. Patients with PIPA can present with recurrent pneumonia and hemoptysis owing to pulmonary hypertension or enlarged collateral vascular structures, or PIPA may be seen as an incidental finding in adults. Although conservative treatment is preferred, transcatheter selective embolization of the enlarged collateral vascular structures and pneumonectomy can be performed because of clinically significant hemoptysis [48, 49].

#### Pulmonary artery sling

Pulmonary artery sling also called the anomalous origin of the left pulmonary artery from the right is an anomaly in which the aberrant left pulmonary artery arises from the posterior aspect of the right pulmonary artery and travels to the left side. While the aberrant left pulmonary artery passes to the left side, it loops around the trachea, forming a sling. Two types of pulmonary artery sling are defined according to the carinal location. The normal location of the carina is at the level of T4-T5 in type 1, while the carina is located inferiorly at the level of T6 with a T-shaped carina in type 2 [1, 3].

**Fig. 19:**
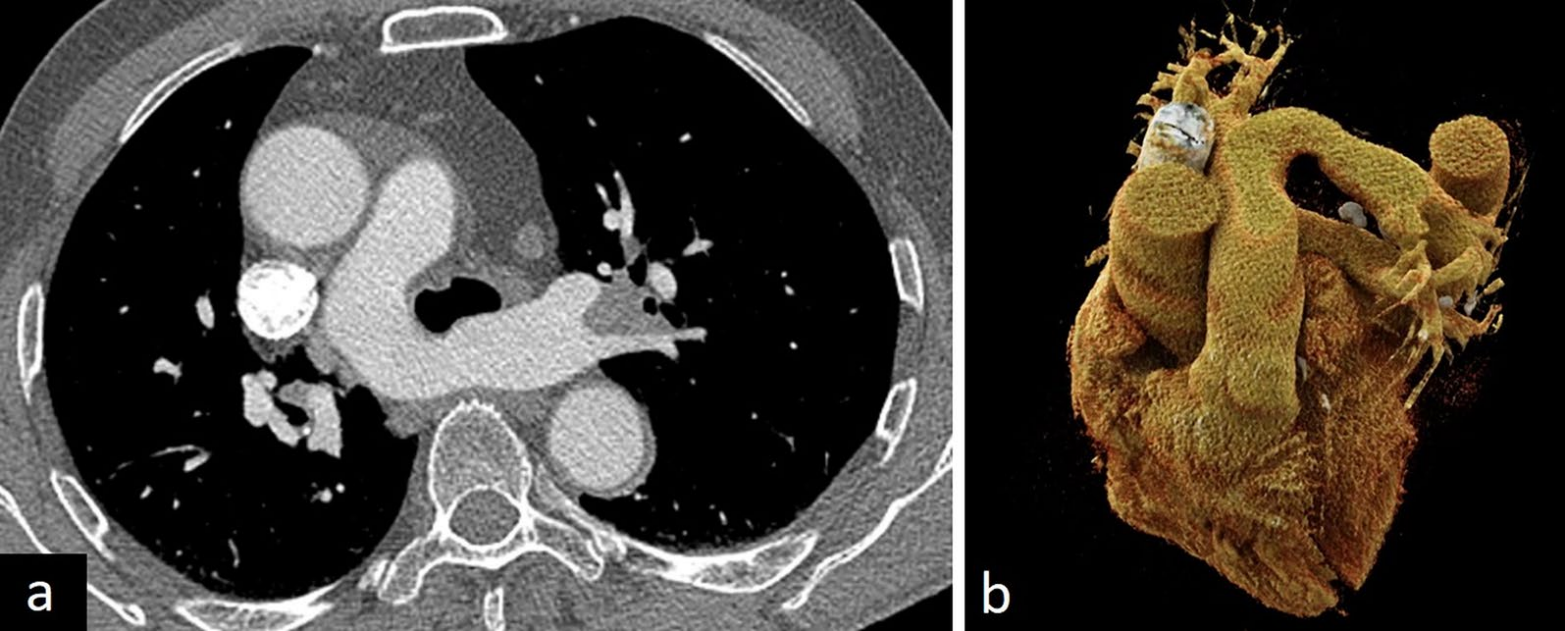
A 71-year-old asymptomatic male with pulmonary artery sling. Contrast-enhanced axial CT image (**a**) and 3D cinematic volume-rendered image (**b**) depict the aberrant left pulmonary artery arising from the right pulmonary artery and crossing to the left hemithorax

Imaging features of pulmonary artery sling vary according to the type. In type 1, posterior tracheal compression and anterior esophageal compression can be depicted on CT images. Hyperinflation of the right lung can also be observed because of the compression of the bronchus. In type 2, distal displacement, long-term stenosis and T-shaped configuration of the trachea and other congenital abnormalities such as congenital cardiovascular and other lung diseases can be seen. In addition to CT, MRI can also accurately show the anomalous origin of the left pulmonary artery from the right [2, 3] (Fig. 19).

Pulmonary artery sling can present with respiratory symptoms such as stridor and difficulty in breathing, although it may be detected incidentally during other radiological examinations. Symptomatic patients can be treated surgically with reimplantation of the anomalous left pulmonary artery and tracheobronchial reconstruction [3, 50].

### Anomalies of the pulmonary vein

Pulmonary venous anatomy is various. The majority of people have four separate pulmonary vein ostia which are derived from the left atrium. Two of the ostia drain the right superior and inferior pulmonary veins, while two of the ostia drain the left superior and inferior pulmonary veins. However, common ostia or different ostial, branching, and drainage patterns can be detected. Depicting the variants of pulmonary vein ostia before an interventional procedure is important to treat atrial fibrillation [51]. Apart from these varied and common variants of pulmonary veins, congenital anomalies of pulmonary veins can be listed as anomalous pulmonary venous drainage and pulmonary venous varix.

#### Anomalous pulmonary venous drainage

**Fig. 20:**
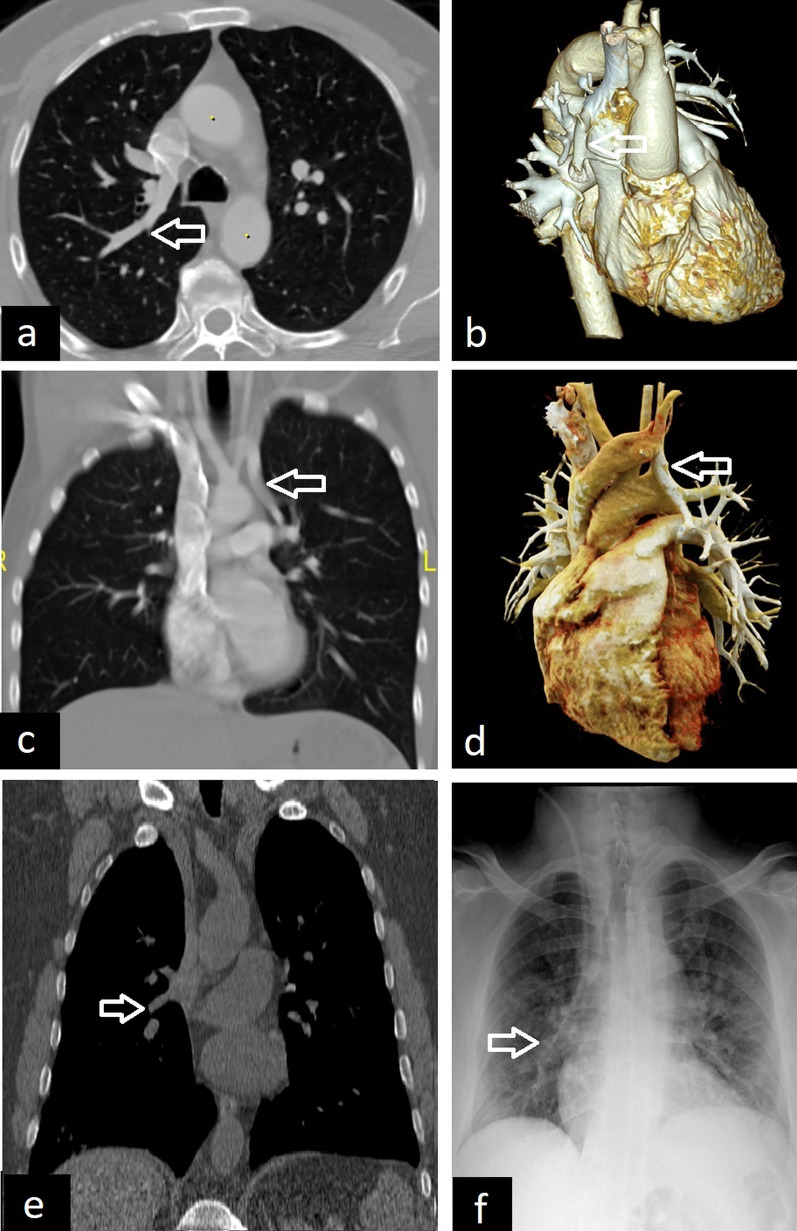
Different examples of partial anomalous pulmonary venous return (PAPVR). Axial CT (**a**) and 3D volume-rendered (**b**) images demonstrate anomalous right upper lobe pulmonary veins draining into the superior vena cava (arrows). The anomalous left upper lobe pulmonary vein draining into the brachiocephalic vein is observed on coronal CT (**c**) and 3D volume-rendered (**d**) images (arrows). An anomalous pulmonary vein is seen on coronal CT image (**e**, arrow) and central venous catheter malposition with the catheter tip extending towards the right lung in the same patient observed on chest X-ray (**f**, arrow)

Anomalous pulmonary venous drainage is divided into two groups; partial anomalous pulmonary venous return (PAPVR) and total anomalous pulmonary venous return (TAPVR). TAPVR is most commonly a cyanotic condition characterized by abnormal drainage of the all pulmonary venous system. Almost all of these patients are diagnosed and corrected in early childhood because of symptoms such as cyanosis and dyspnea [51, 52]. PAPVR is an anomaly in which some of the pulmonary veins, one or more (but not all), drain into the systemic circulation. It can occur in adults with a prevalence of 0.1%–0.2% [51]. Right-sided PAPVR is usually found in children and is more often related to an atrial septal defect. Left-sided PAPVR is more common in adults. The most common pattern of right-sided PAPVR is the right superior pulmonary vein draining directly into the right atrium or superior vena cava. The most common type of left-sided PAPVR is the left pulmonary vein draining into the left brachiocephalic vein via the cardinal vein, which is also known as the vertical vein. Less common types of PAPVR draining into any systemic circulation can also be observed [51]. CT, 3D reconstruction images, and MRA can demonstrate PAPVR in detail (Fig. 20).

PAPVR can be asymptomatic and found incidentally in adults. The abnormal position of a central venous catheter on the chest X-ray can also be a clue for the diagnosis of PAPVR (Fig. 20). PAPVR can rarely cause adult-onset pulmonary arterial hypertension. PAPVR and the associated atrial septal defect may cause left-to-right shunt and right heart remodeling [51, 53].

#### Pulmonary venous varix

Pulmonary venous varix, which is the focal dilatation of the pulmonary vein, can occur as congenital or acquired due to post-traumatic or increased pulmonary vein pressure. Although it is usually seen near the pulmonary vein ostium and on the right lower side of the thorax, peripheral pulmonary vein varix can also occur [3, 10, 51].

**Fig. 21:**
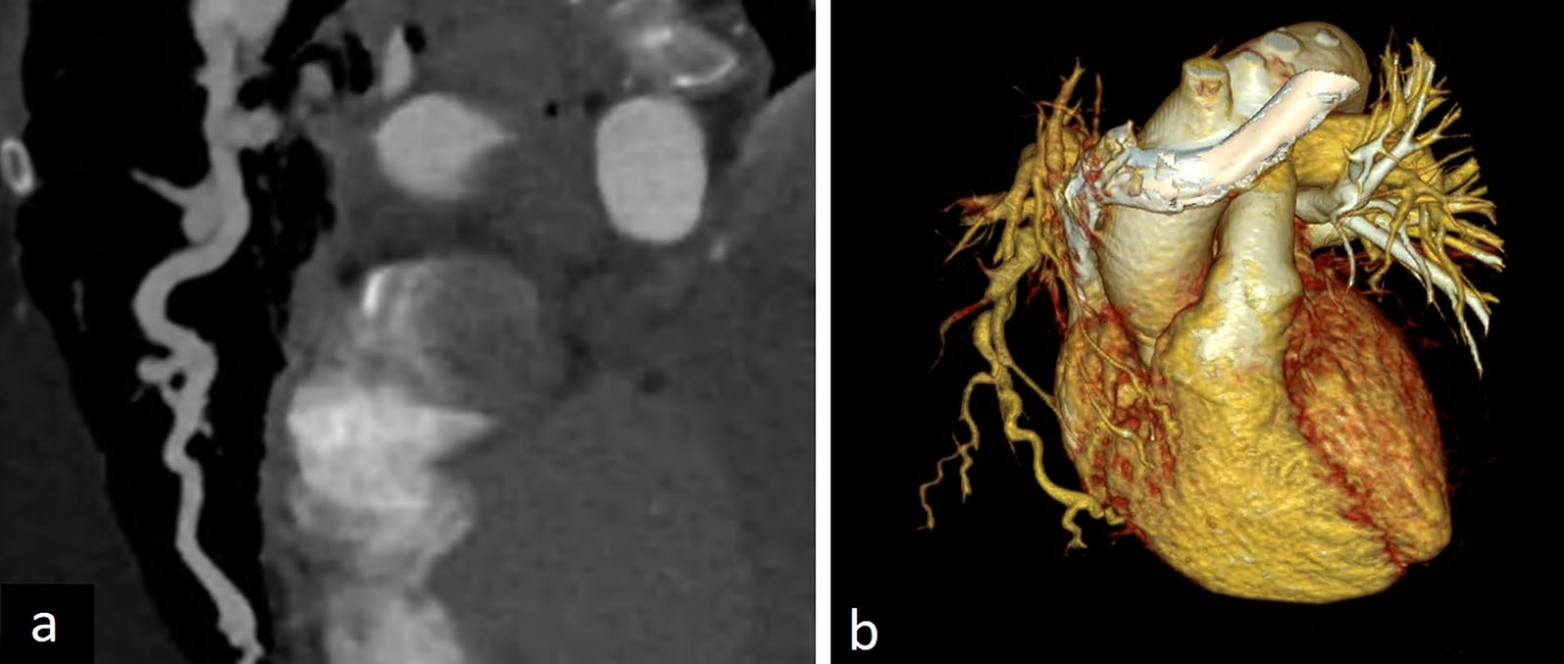
Pulmonary varix. Curved multiplanar reconstruction (**a**) and 3D volume-rendered CT image (**b**) demonstrate pulmonary varix in a 73-year-old female

The characteristic radiological findings of pulmonary varix can be seen on CT or MR as a focal enlargement of a pulmonary vein. It appears wider than the adjacent pulmonary vein. Pulmonary arteriovenous malformation (PAVM) is considered in the differential diagnosis of pulmonary varix. Criteria including normal pulmonary artery tree, varix drainage to the left atrium without shunt between pulmonary and systemic circulations, late drainage of varix compared to the other pulmonary veins, and tortuosity only in the proximal section of pulmonary varix help in the differentiation of pulmonary varix from a PAVM and definitive diagnosis. The absence of a feeding artery in pulmonary varix can also help to distinguish it from PAVM [51, 54] (Fig. 21).

Pulmonary varix is usually incidentally reported and asymptomatic and does not require treatment. However, they can be resected surgically when they cause symptoms and complications such as cough, palpitation, dyspnea, cerebral embolism, rupture, and hemoptysis [3, 51, 55].

### Pulmonary arteriovenous malformation (PAVM)

PAVM results from direct communication between the pulmonary artery and vein. Although most PAVMs are congenital and may be related to hereditary hemorrhagic telangiectasia, they may occur as a result of prior congenital cyanotic cardiac surgery, chronic liver disease, or atypical lung infection such as tuberculosis and actinomycosis [56–58]. Most PAVMs are found in the lower lobes, although they can occur in any lobe. They can be seen as single or multiple lesions involving one or both lungs [2].

**Fig. 22:**
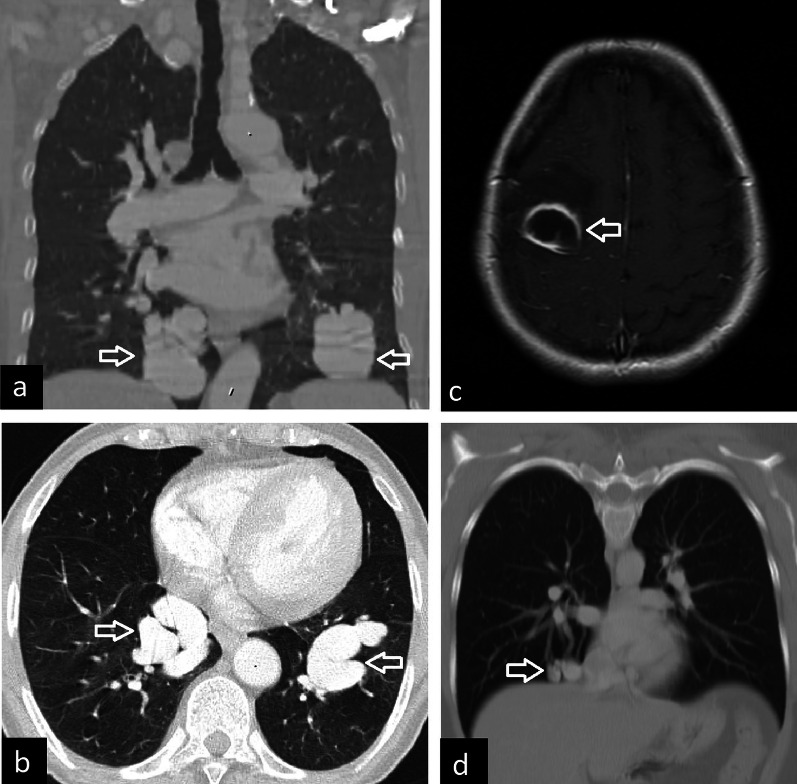
Pulmonary arteriovenous malformation (PAVM). Coronal (**a**) and axial (**b**) CT images show bilateral PAVM in a 69-year-old female (arrows). Contrast-enhanced T1-weighted MRI (**c**, arrow) depicts the brain abscess due to paradoxical embolization with ring enhancement and surrounding edema in a 36-year-old female. PAVM in the right lower lobe of the same patient is observed on coronal chest CT image (**d**, arrow)

Contrast-enhanced CT and 3D reconstructions can depict PAVM in detail. A large single sac, a mass of enlarged vascular structures, and direct communication between artery and vein can be listed as radiological findings of PAVM (Fig. 22). Mural thrombi or calcifications may also occasionally be seen. PAVM with a single feeding artery and a single draining vein is defined as simple, while PAVM with 2 or more feeding arteries or draining veins is defined as complex PAVM. Although PAVM is usually supplied by pulmonary arteries and drains to the left atrium, communication with systemic arterial or venous circulation can also be seen [4].

Although small and single PAVMs can be asymptomatic, they can present with dyspnea, cyanosis, and clubbing due to high right-to-left shunt. Moreover, patients with PAVM may develop serious complications including pulmonary hemorrhage because of rupture of PAVM or stroke and brain abscess due to paradoxical embolization [2] (Fig. 22). The main treatments of PAVM are endovascular coil embolization or balloon occlusion [59]. Surgical treatment can also be performed.

### Anomalies of the bronchial artery

**Fig. 23:**
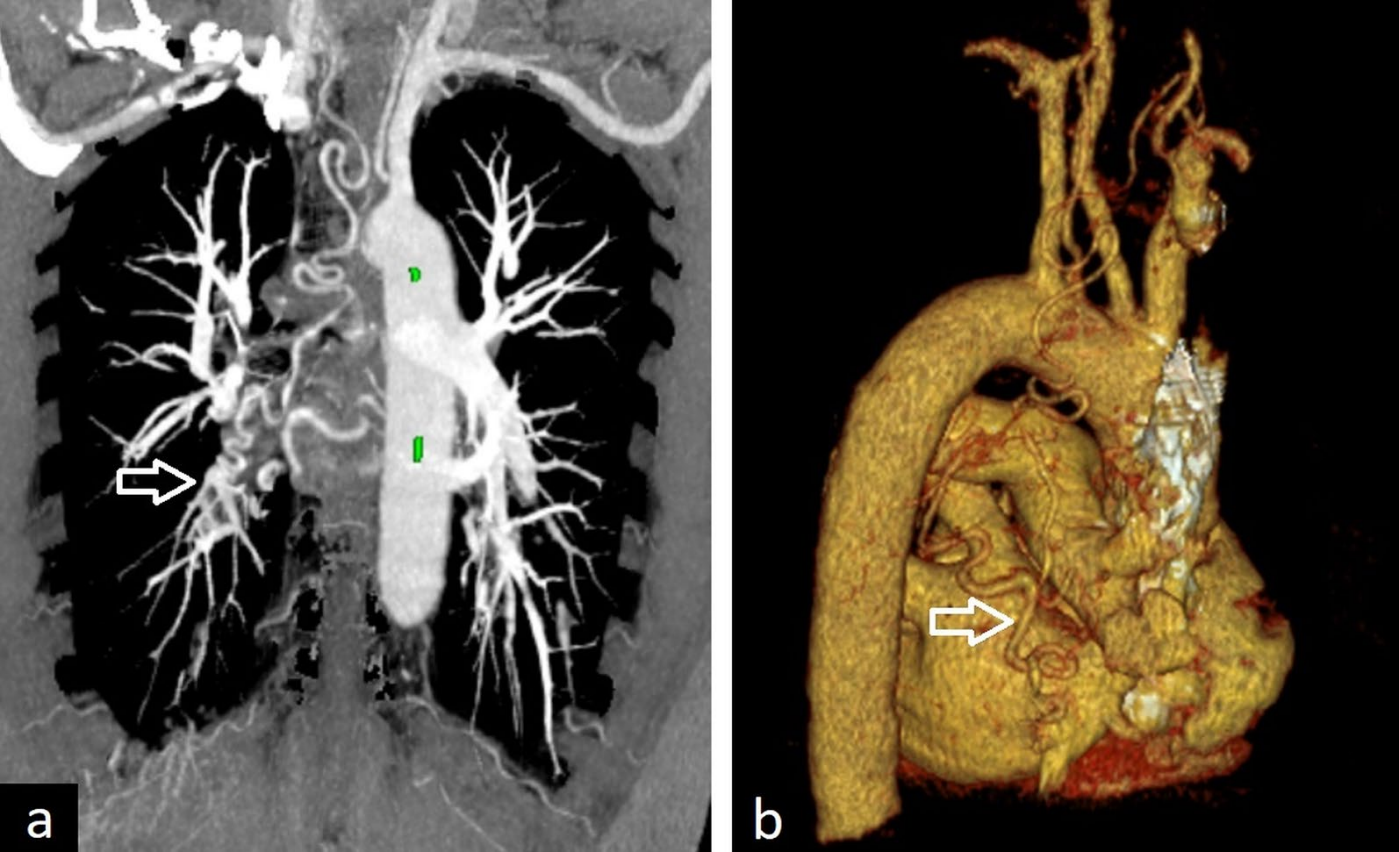
Abnormal dilated bronchial arteries. Maximum intensity projection (MIP) (**a**) and 3D volume-rendered (**b**) images show ectopic dilated bronchial artery originating from the right subclavian artery (arrows) in a 63-year-old male with bronchiectasis

The bronchial arteries generally arise from the proximal descending thoracic aorta. They are named orthotopic when they arise between the T5 and the T6 vertebral bodies. They are named ectopic when they originate elsewhere in the aorta or from other vascular structures including the subclavian artery, brachiocephalic trunk, internal mammary artery, and coronary artery. Left bronchial arteries usually originate directly from the aorta, while right bronchial arteries share their origin with another artery, such as an intercostal artery. The normal caliber of the bronchial arteries is less than 2 mm at their origin [60, 61]. Abnormal dilated bronchial arteries can be visualized on CT (Fig. 23).

Bronchial arteriovenous malformation (BAVM) is an uncommon congenital condition that can cause a left-to-left or left-to-right extra-cardiac shunt. [60, 62]. They can also be acquired as a result of infectious or inflammatory lung diseases, tumors, and trauma. CT findings of BAVM are tortuous, enlarged bronchial artery with focal aneurysm and abnormal connection with a pulmonary artery or vein.

Patients with BAVM are usually asymptomatic, but they can present with massive hemoptysis and require embolization [60].

## Combined anomalies

Combined congenital anomalies of the lung refer to anomalies that have both abnormal parenchyma and vascularity. They can be listed as hypogenetic lung (scimitar), bronchopulmonary sequestration, and mixed lesions.

### Hypogenetic lung (scimitar)

Hypogenetic lung syndrome, also called congenital pulmonary venolobar syndrome or scimitar, is a rare congenital anomaly that almost always affects the right lung. The main features of scimitar syndrome are anomalous venous return which has a scimitar-like appearance, cardiac dextroversion, hypoplasia of the right lung, pulmonary artery, and anomalous systemic arterial supply to the right lung. Other associated anomalies such as atrial and ventricular septal defects and diaphragmatic abnormalities can be seen in up to 25% of patients with scimitar syndrome [1, 63].

**Fig. 24:**
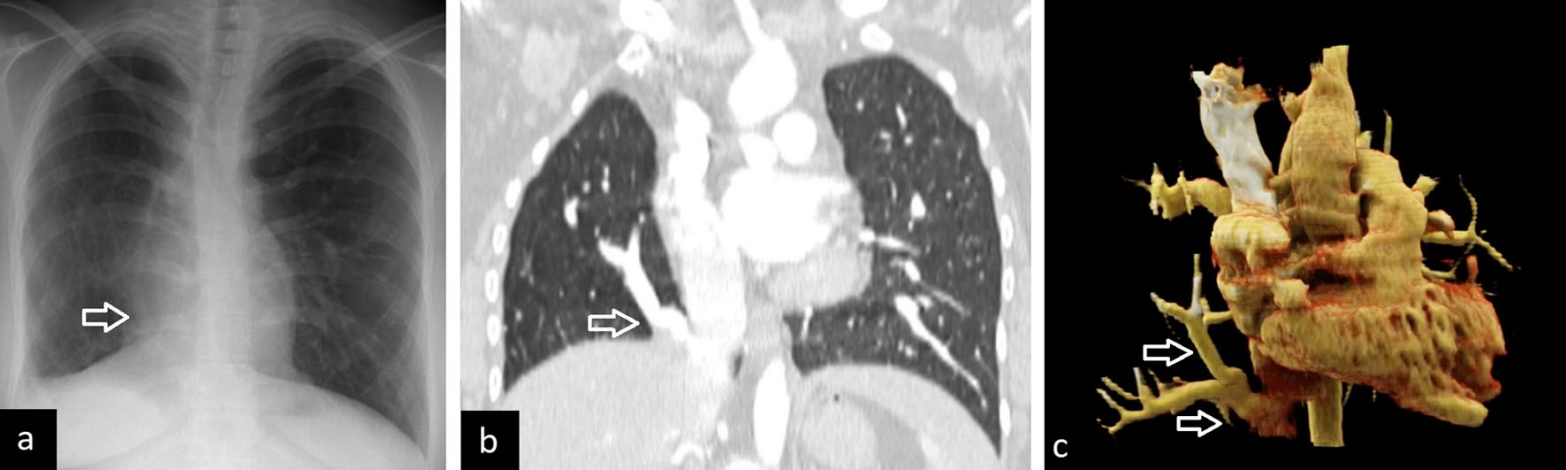
A 25-year-old female with scimitar syndrome. Chest X-ray (**a**) and coronal CT image (**b**) demonstrate right lung hypoplasia and curvilinear tubular opacity joining the inferior vena cava above the medial aspect of the right hemidiaphragm (arrows). 3D reconstruction image obtained with the cinematic volume-rendering-technique shows (**c**) the way of the anomalous pulmonary vein (arrows)

The typical radiological characteristics are small, hyperlucent hypoplastic right lung with associated dextroversion of the heart, mediastinal shift to the right, hyperinflation of the contralateral side, and a curvilinear anomalous vein with a scimitar-like appearance. The scimitar vein usually drains into the inferior vena cava, but may drain into the right atrium, superior vena cava, azygos vein, portal vein, and hepatic vein [2, 63, 64] CT images, especially 3D reconstruction images can demonstrate both anomalous venous return and anomalous arterial supply (Fig. 24). CT can also show abnormal lung parenchyma including bronchiectasis, consolidation, bronchial branching, and abnormal lung lobation.

Although scimitar syndrome can remain asymptomatic, it can also present with recurrent right pulmonary infections and is associated with the left-to-right shunt. Asymptomatic patients without pulmonary hypertension may be observed. Symptomatic patients are treated in two steps including ligation or embolization of the systemic artery, reimplantation of the scimitar vein into the left atrium, and excision of the recurrently infected lung [4]. CT also aids in assessing postoperative complications such as thrombosis and stenosis of reimplanted vascular structures.

### Bronchopulmonary sequestration

Pulmonary sequestration is an anomaly presenting with dysplastic non-functional pulmonary tissue that is not related to the normal tracheobronchial tree and is supplied from systemic circulation [2–4, 10, 65]. Pulmonary sequestrations are divided into two types as intralobar and extralobar. Extralobar sequestrations have their own pleura, drain into the systemic venous circulation, show association with up to 60% of other congenital anomalies, and most commonly present in childhood [65, 66]. Intralobar sequestrations are anomalies that can be seen in adulthood. They usually drain into the ipsilateral pulmonary venous system and do not have their own pleura, but share pleural investment with adjacent pleura.

**Fig. 25:**
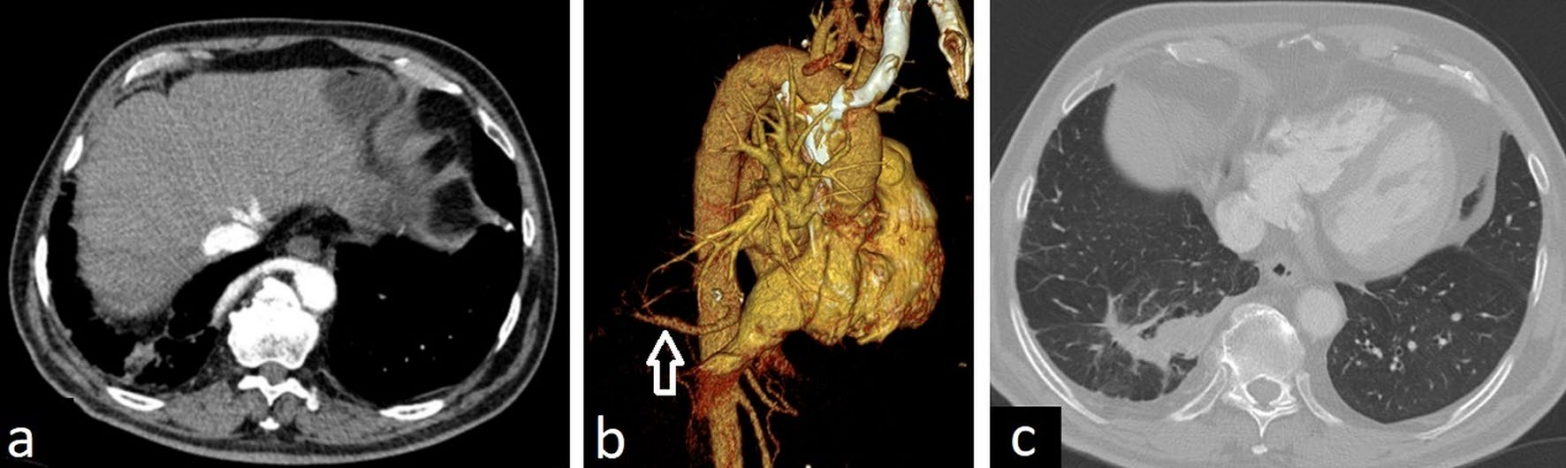
A 67-year-old asymptomatic male with sequestration. Contrast-enhanced axial CT image in the mediastinal window (**a**) and 3D volume-rendered reconstruction image (**b**) demonstrate the aberrant artery originating from the descending thoracic aorta (arrows). Axial CT image in the lung window (**c**) depicts right lower lobe opacity corresponding to the sequestration

**Fig. 26:**
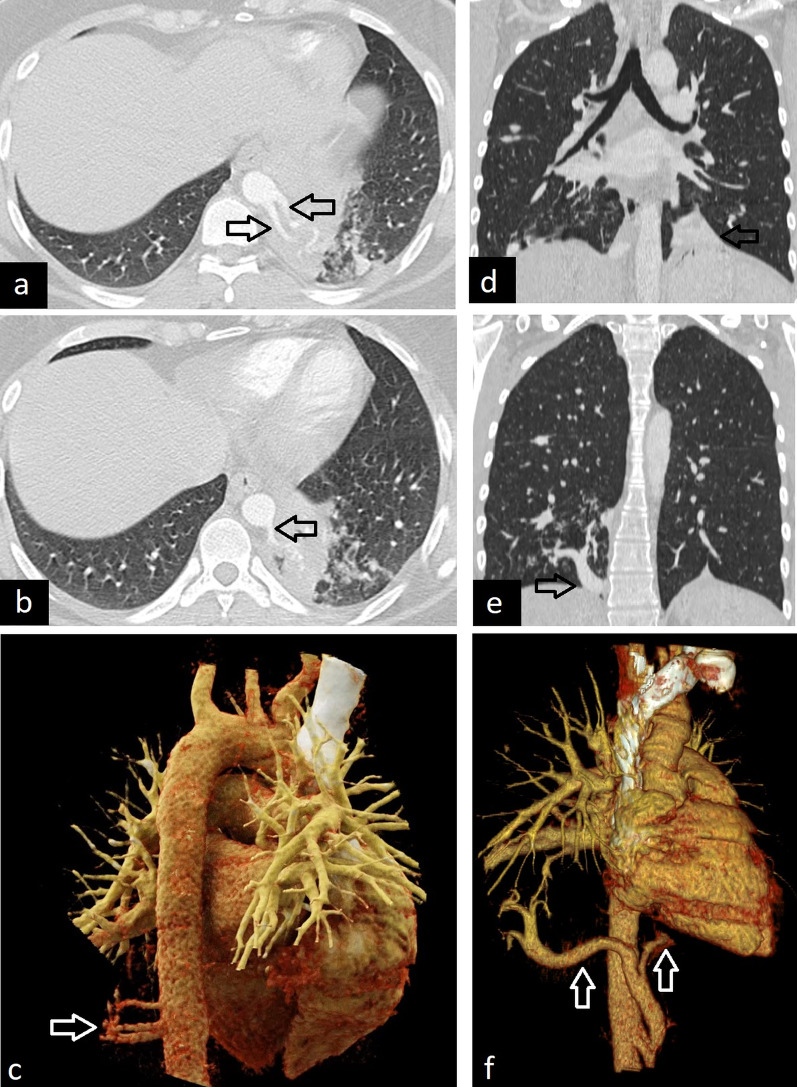
Different examples of pulmonary sequestrations. Axial CT images (**a**, **b**) and 3D cinematic volume-rendered reconstruction image (**c**) show three aberrant arteries which arise from the descending thoracic aorta and feed a consolidated area in the left lower lobe in a 25-year-old female with sequestration who presented with dyspnea (arrows). Coronal CT images (**d**, **e**) and 3D volume-rendered reconstruction (**f**) image demonstrate bilateral sequestrations with aberrant arteries arising from the abdominal aorta in a 22-year-old asymptomatic woman (arrows)

MR and CT angiography especially 3D reconstructions and MPR images can depict anomalous systemic arterial supply in sequestrations (Fig. 25). Bronchopulmonary sequestration can be supplied by more than one artery, and it can also occur bilaterally (Fig. 26). Atherosclerotic changes are commonly seen on CT images. Assessment of the lung parenchyma can be better depicted with the high-resolution CT. The most commonly involved area is the posterior basal segment of the left lower lobe. The sequestration can be seen as consolidation, atelectasis, or as a solid lesion mimicking a tumor. Cystic areas and air-fluid levels may occur within sequestration. Due to the cystic appearance, CPAM, abscess, and cavitating malignancy should be considered in the differential diagnosis.

Intralobar sequestrations usually present with recurrent infections, although they can also be asymptomatic and incidentally detected. For symptomatic patients, surgical excision is usually done. In asymptomatic patients, elective surgical excision is also usually preferred due to the risks of recurrent infection, hemorrhage, and possible risk of malignancy [2, 4]. Demonstrating vascular structures in detail on CT before surgery is essential to prevent surgical complications and obtain successful surgical outcomes.

### Mixed lesions

**Fig. 27:**
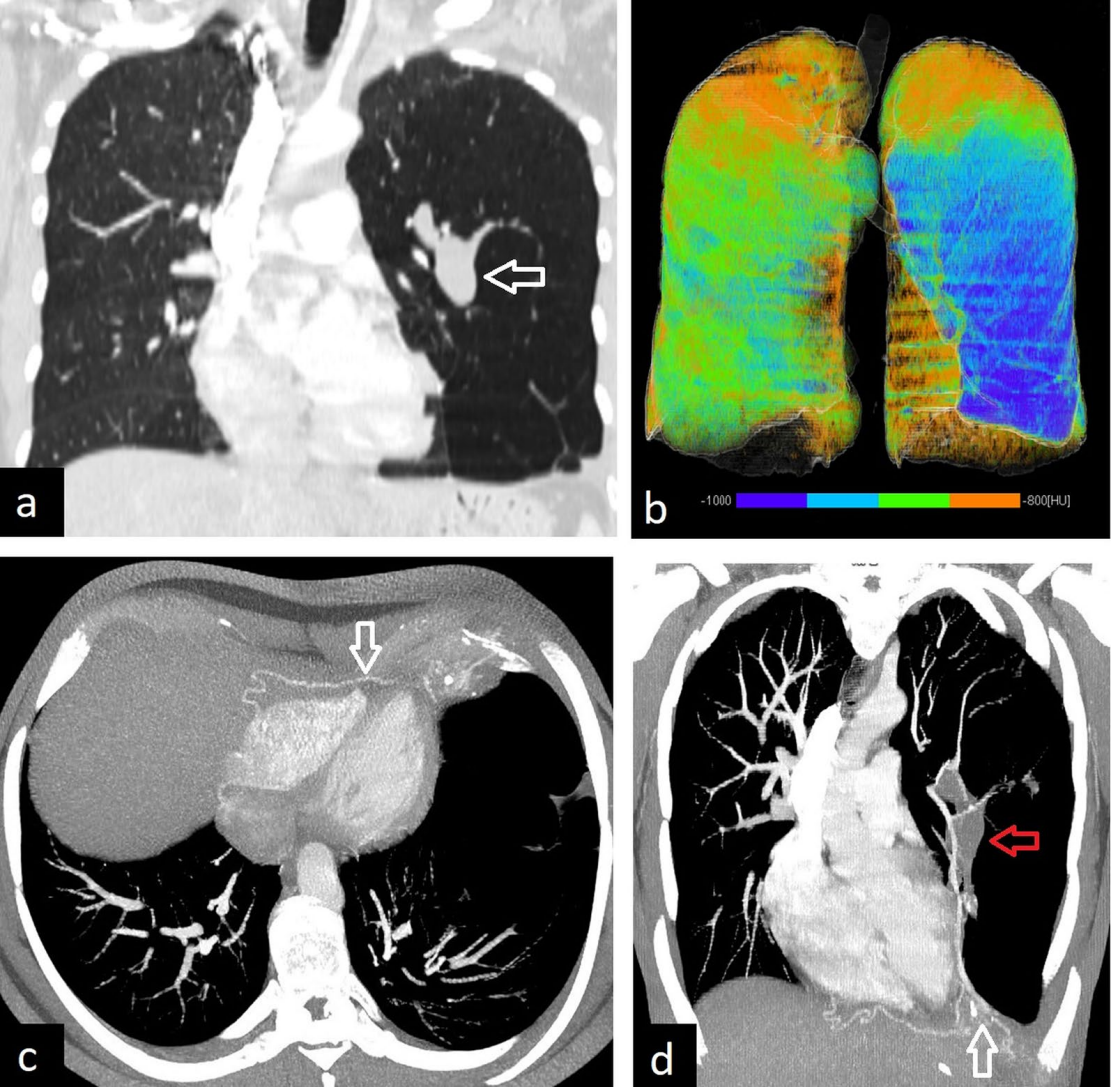
Mixed lesions-pulmonary sequestration and bronchial atresia in a 45-year-old asymptomatic female. Coronal CT image shows bronchial atresia with the finger-in-glove appearance (**a**, arrow). Hyperinflation of the obstructed lung area is observed on both coronal (**a**) and quantitative 3D reconstruction CT images (blue area) (**b**). Axial (**c**) and coronal (**d**) maximum intensity projection (MIP) images demonstrate pulmonary sequestration supplied from an aberrant artery arising from the right inferior phrenic artery (arrows). Bronchial atresia is also seen on the coronal MIP image (red arrow)

**Fig. 28:**
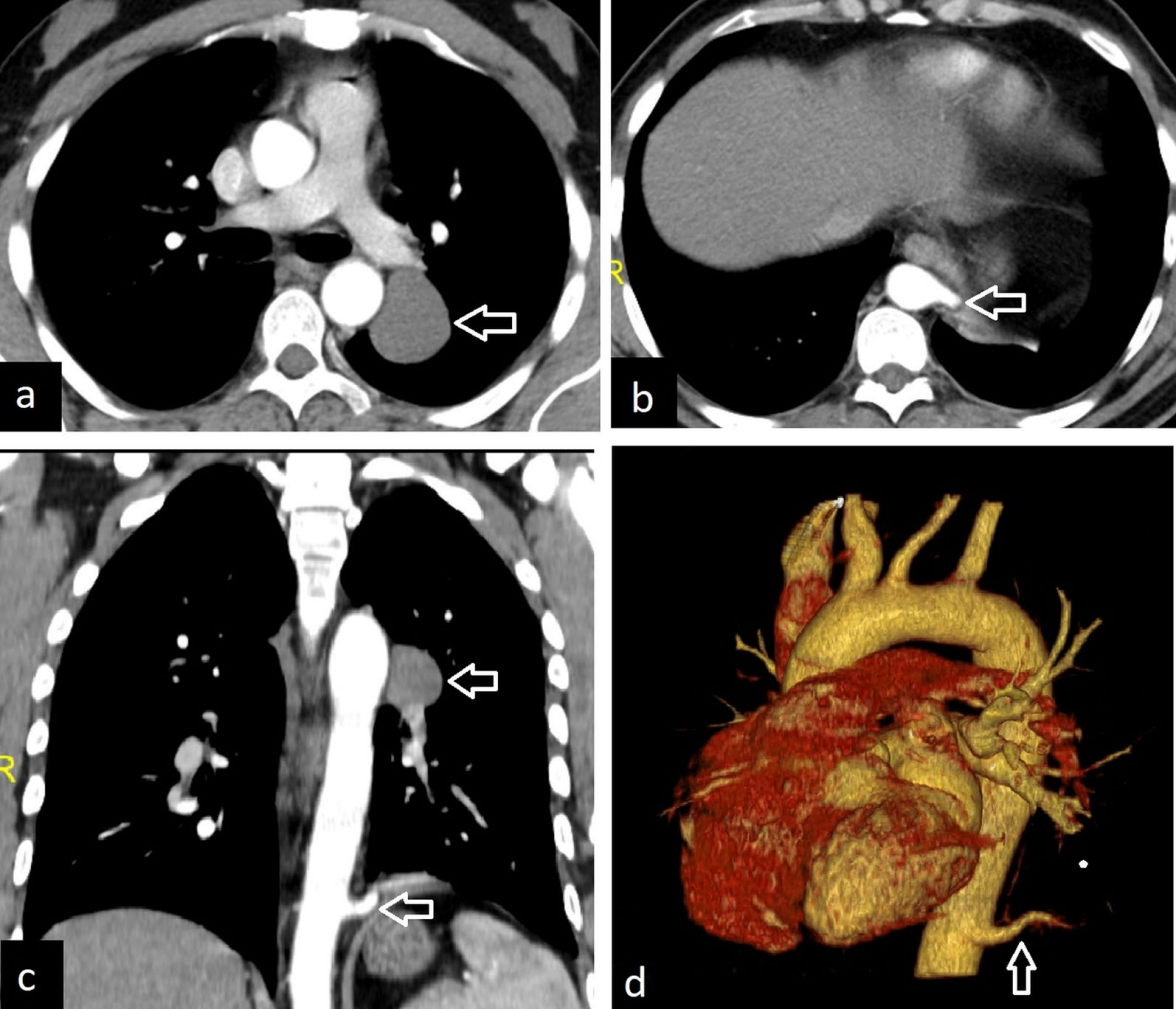
Mixed lesions-pulmonary sequestration and bronchogenic cyst in a 38-year-old male who presented with dyspnea. Contrast-enhanced axial CT images show bronchogenic cyst (**a**, arrow) and aberrant artery arising from the descending aorta and feeding the left lower lobe sequestration (**b**, arrow). Coronal CT image (**c**) shows both bronchial cyst and aberrant artery of sequestration (arrows). 3D volume-rendered CT image (**d**) shows the aberrant artery (arrow)

**Fig. 29:**
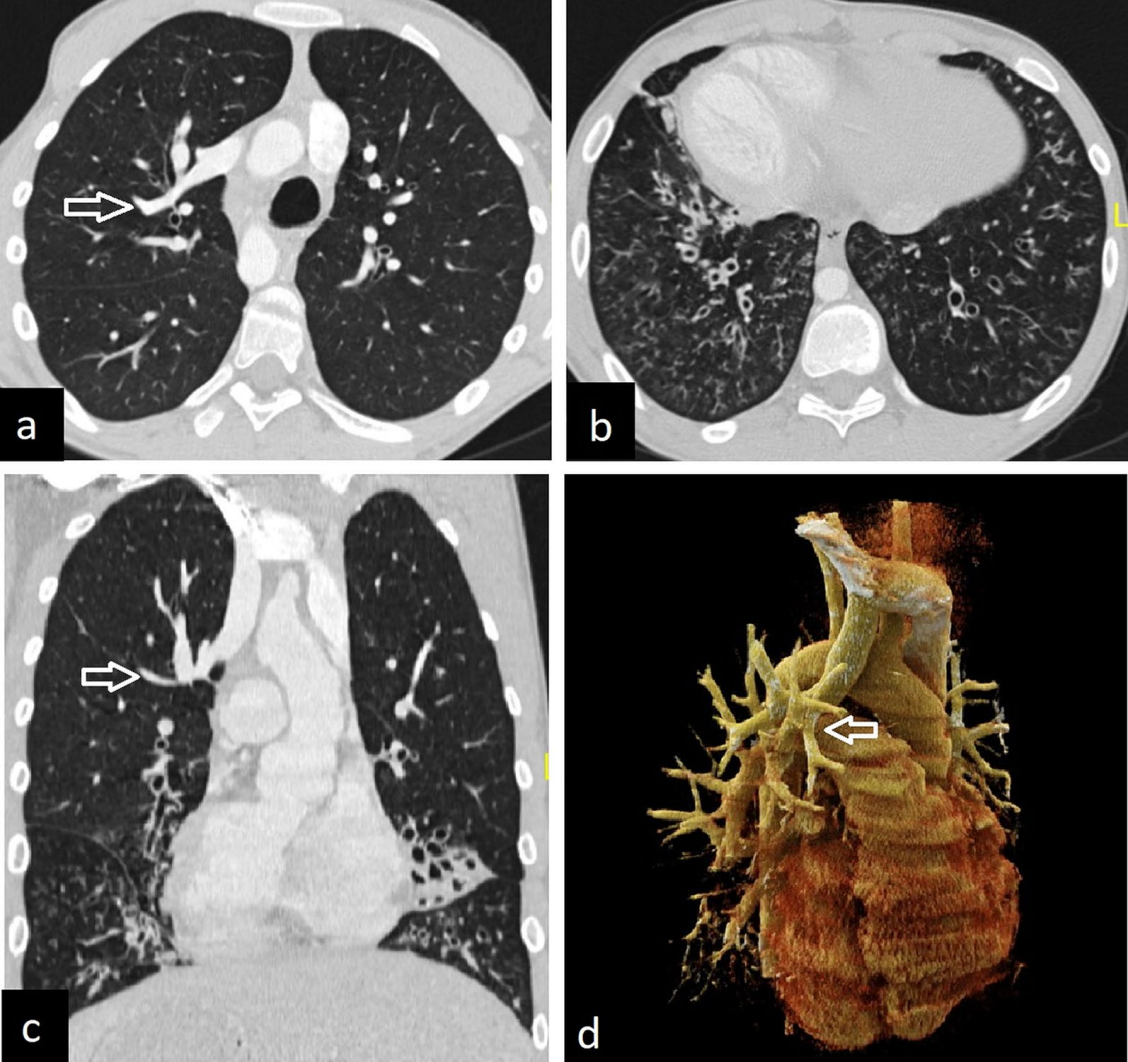
Mixed lesions-Kartagener syndrome and partial anomalous pulmonary venous return (PAPVR) in a 20-year-old man. Axial (**a**) and coronal (**c**) CT images show anomalous right upper lobe pulmonary veins draining into the right brachiocephalic vein. Bronchiectasis, bronchial wall thickening, and centrilobular opacities predominantly affecting lower lobes of the lungs are seen on axial CT image (**b**). Coronal CT image (**c**) demonstrates both PAPVR (arrow) and bronchiectasis. 3D volume-rendered reconstruction image (**d**) shows PAPVR (arrow)

More than one congenital lung disease can be observed together. Mixed bronchopulmonary sequestration and CPAM are defined as hybrid lesions and almost all of these have been reported in children [67, 68]. However, bronchopulmonary sequestration can also concur with other congenital lung diseases in adults such as bronchogenic cyst and bronchial atresia. Except for bronchopulmonary sequestration, various congenital lung diseases can occur together as mixed lesions in adults (Figs. 27, 28, 29).

## New applications that can be used in the diagnosis of congenital lung diseases

Advances in CT technology provide better images with thinner slice thickness, MPR images with similar image quality to axial CT images, maximum intensity projection (MIP) images, 3D volume rendering images with great anatomical detail, and quantitative 3D reconstruction images.

MIP images are created by the highest voxel attenuation values from the volume of CT data. MIP images can be used for both detection of lung nodules and assessing the vessels. 3D volume rendering and MIP images can be very helpful in congenital lung diseases by showing the size and location of the vessels in more detail. Also, they provide better visualization of anatomy for surgical treatment planning. In addition to vessel assessment, volume-rendered images can ensure virtual bronchoscopy to evaluate airways.

Minimum intensity projection (MinIP) images are constituted by low-density structures in a volume of CT data. So, MinIP images display the most hypodense structures of the volume and help the detection and localization of mosaic attenuation and cystic lung diseases. Quantitative 3D reconstruction CT images are produced using dedicated software according to the HU values of the entire CT images and show various density levels with different colors [69, 70]. Both quantitative 3D reconstruction images and MinIP images demonstrate congenital cystic lung diseases and air trapping areas as well as high-density areas.

CT angiography (CTA) and subsequently obtained 3D or MIP images can demonstrate abnormal vascular structures and can help the diagnosis of vascular and combined anomalies of congenital lung diseases. With the improvement of CT, CTA, and dual-energy CT (DECT), pulmonary perfusion can be evaluated. DECT allows differentiating compositions of tissues based on image acquisition at two different energy levels. In addition to lesion characterization, iodine maps showing the pulmonary perfusion can be created from DECT. DECT perfusion mapping is usually used for the assessment of pulmonary embolism. It can be also performed for both showing vascular anomalies and combined anomalies such as bronchopulmonary sequestration. On the other hand, perfusion defects because of parenchymal diseases such as emphysema can be depicted on DECT perfusion mapping [71].

Chest CT can be acquired with a very low radiation dose due to new CT detector and reconstruction technologies [72, 73]. But, lung MRI can be preferred in young patients who need repetitive follow-up imaging. Furthermore, soft tissue contrast resolution of MRI is better and differentiation between solid lesion and cyst with high-density content can be done via MRI. This feature helps the diagnosis of bronchogenic cyst. With the innovations of MRI technology, non-contrast thoracic MR angiography (MRA) can also be used in showing vascular abnormalities. This advantage of MRI avoids both extra cost and nephrogenic systemic fibrosis due to gadolinium usage. Gradient echo sequences such as balanced steady-state free precession can depict thoracic vascular structures with similar diagnostic quality [74, 75].

Ultra-Short Echo Time (UTE) MRI is an ideal technique for lung imaging because of its minimized echo time (< 100 µsec) and higher signal-to-noise ratio compared to a conventional MRI sequence with a short TE. The assessment of lung and mediastinal abnormalities with UTE MRI has been found as good as CT. In addition to the morphological assessment of the lungs, novel UTE sequences offer simultaneously regional functional information. Functional analysis of 3D UTE MRI can provide the evaluation of ventilation inhomogeneity and hyperinflation in patients with congenital lung diseases such as cystic fibrosis [76–78]. Lung function can also be assessed with dynamic contrast-enhanced (DCE) MRI, oxygen-enhanced (OE) MRI, arterial spin labeling (ASL) and, phase-resolved functional lung (PREFUL) [79, 80]. DCE-MRI is the most well-known and well-established method of lung perfusion. The main principle of DCE-MRI is time-resolved data acquisition following intravenous bolus injection of contrast agent [81, 82]. OE-MRI can be used for ventilation imaging due to the paramagnetic property of oxygen. Dissolved molecular oxygen and deoxyhemoglobin reduce the relaxation time of T1 and increase signal intensity on T1 images. So, the regional oxygen transfer which is affected by ventilation, perfusion, and diffusion capacity of pulmonary parenchyma can be shown [79, 83, 84]. ASL-MRI is a non-contrast method for the evaluation of pulmonary ventilation and perfusion. It is based on labeled water protons in arterial blood. The blood is labeled by radiofrequency pulses to invert the magnetization. Also, control images are obtained from the same anatomical region. A perfusion map is acquired by subtraction of control and labeled images [85, 86]. PREFUL is another valuable method that can show perfusion and ventilation in different diseases during free breathing and without the application of a contrast agent. PREFUL is a further development of Fourier decomposition (FD) post-processing technique. FD is based on the registration of dynamic images of the lung, in which periodic signal changes related to perfusion and ventilation can be spectrally separated and subsequently evaluated [79, 87–89].

## Conclusion

The diagnosis of congenital lung diseases in adults can be difficult because of their rarity, non-specific symptoms, and resemblance to other lung diseases. However, congenital lung diseases may have distinctive radiological findings. Besides conventional CT and MRI images, MPR, 3D reconstructions, quantitative 3D reconstruction images, and DECT can help depict congenital lung diseases. Novel MRI techniques such as ultra-short echo time (UTE), arterial spin labeling (ASL), and phase-resolved functional lung (PREFUL) can provide functional information. Familiarity with imaging findings of different types of congenital lung diseases will enable the correct diagnosis and management of these diseases. Furthermore, the correct diagnosis can prevent potential complications and unnecessary worry of patients as congenital lung abnormalities may mimic neoplasms in adults.

## Data Availability

Data sharing is not applicable. Because this article has no datasets that were constituted or analyzed during the current study.

## Abbreviations

3D: Three-dimensional
ASL: Arterial spin labeling
BAVM: Bronchial arteriovenous malformation
BHDS: Birt–Hogg–Dubé syndrome
CLH: Congenital lobar hyperinflation
CPAM: Congenital pulmonary airway malformation
CT: Computed tomography
CTA: Computed tomography angiography
DCE: Dynamic contrast-enhanced
DECT: Dual-energy computed tomography
FD: Fourier decomposition
LAM: Lymphangioleiomyomatosis
MinIP: Minimum intensity projection
MIP: Maximum intensity projection
MPR: Multi-planar reconstruction
MRA: Magnetic resonance angiography
MRI: Magnetic resonance imaging
NF 1: Neurofibromatosis type 1
OE: Oxygen-enhanced
PAPVR: Partial anomalous pulmonary venous return
PAVM: Pulmonary arteriovenous malformation
PIPA: Proximal interruption of the pulmonary artery
PREFUL: Phase-resolved functional lung
TAPVR: Total anomalous pulmonary venous return
TSC: Tuberous sclerosis complex
UTE: Ultra-short echo time

## References

[1] Zylak CJ, Eyler WR, Spizarny DL, Stone CH (2002) Developmental lung anomalies in the adult: radiologicpathologic correlation. Radiographics S25-43 10.1148/radiographics.22.suppl_1.g02oc26s25 12376599

[2] Lee EY, Boiselle PM, Cleveland RH (2008). Multidetector CT evaluation of congenital lung anomalies. Radiology.

[3] Thacker PG, Rao AG, Hill JG, Lee EY (2014). Congenital lung anomalies in children and adults: current concepts and imaging findings. Radiol Clin N Am.

[4] Trotman-Dickenson B (2015). Congenital lung disease in the adult: guide to the evaluation and management. J Thorac Imaging.

[5] Muensterer O, Abellar R, Otterburn D, Mathew R (2015). Pulmonary agenesis and associated pulmonary hypertension: a case report and review on variability, therapy, and outcome. Eur J Pediatr Surg Rep.

[6] Gupta K, Taneja D, Aggarwal M, Gupta R (2017). Left upper lobar agenesis of lung: a rare case report. Lung India.

[7] Katsenos S, Antonogiannaki E-M, Tsintiris K (2014). unilateral primary lung hypoplasia diagnosed in adulthood. Respir Care.

[8] Roy PP, Datta S, Sarkar A, Das A, Das S (2012). Unilateral pulmonary agenesis presenting in adulthood. Respir Med Case Rep.

[9] Biyyam DR, Chapman T, Ferguson MR, Deutsch G, Dighe MK (2010). Congenital lung abnormalities: embryologic features, prenatal diagnosis, and postnatal radiologic-pathologic correlation. Radiographics.

[10] Lee EY, Dorkin H, Vargas SO (2011). Congenital pulmonary malformations in pediatric patients: review and update on etiology, classification, and imaging findings. Radiol Clin N Am.

[11] Priest JR, Hill DA, Williams GM (2006). Type I pleuropulmonary blastoma: a report from the International Pleuropulmonary Blastoma Registry. J Clin Oncol.

[12] Ozcan C, Celik A, Ural Z, Veral A, Kandiloglu G, Balik E (2001). Primary pulmonary rhabdomyosarcoma arising within cystic adenomatoid malformation: a case report and review of the literature. J Pediatr Surg.

[13] Federici S, Domenichelli V, Tani G (2001). Pleuropulmonary blastoma in congenital cystic adenomatoid malformation: report of a case. Eur J Pediatr Surg.

[14] Odev K, Guler I, Altinok T, Pekcan S, Batur A, Ozbiner H (2013). Cystic and cavitary lung lesions in children: radiologic findings with pathologic correlation. J Clin Imaging Sci.

[15] Kwon YS, Koh WJ, Han J (2007). Clinical characteristics and feasibility of thoracoscopic approach for congenital cystic adenomatoid malformation in adults. Eur J Cardiothorac Surg.

[16] Pike D, Mohan S, Ma W, Lewis JF, Parraga G (2015). Pulmonary imaging abnormalities in an adult case of congenital lobar emphysema. J Radiol Case Rep.

[17] Çalışkan T, Okutan O, Çiftçi F, Kartaloğlu Z, Taş D, Demirer E (2014). Congenital lobar emphysema diagnosed in adult age: a case report. Eurasian J Pulmonol.

[18] Daccord C, Nicod LP, Lazor R (2017). Cystic lung disease in genetic syndromes with deficient tumor suppressor gene function. Respiration.

[19] Lee JE, Cha YK, Kim JS, Choi JH (2017). Birt–Hogg–Dube syndrome: characteristic CT findings differentiating it from other diffuse cystic lung diseases. Diagn Interv Radiol.

[20] McCormack FX, Gupta N, Finlay GR (2016). Official American Thoracic Society/Japanese Respiratory Society Clinical Practice Guidelines: lymphangioleiomyomatosis diagnosis and management. Am J Respir Crit Care Med.

[21] Tanrivermis Sayit A, Elmali M, Saglam D, Celenk C (2016). The diseases of airway-tracheal diverticulum: a review of the literature. J Thorac Dis.

[22] Zamora AC, Collard HR, Wolters PJ, Webb WR, King TE (2007). Neurofibromatosis-associated lung disease: a case series and literature review. Eur Respir J.

[23] Green DB, Restrepo CS, Legasto AC, Bang TJ, Oh AS, Vargas D. Imaging of the rare cystic lung diseases. Curr Probl Diagn Radiol. 2021. 10.1067/j.cpradiol.2021.02.003 33618900

[24] Devine MS, Garcia CK (2012). Genetic interstitial lung disease. Clin Chest Med.

[25] Shanmugam G (2005). Adult congenital lung disease. Eur J Cardiothorac Surg.

[26] Aktogu S, Yuncu G, Halilcolar H, Ermete S, Buduneli T (1996). Bronchogenic cysts: clinicopathological presentation and treatment. Eur Respir J.

[27] Coselli MP, de Ipolyi P, Bloss RS, Diaz RF, Fitzgerald JB (1987). Bronchogenic cysts above and below the diaphragm: report of eight cases. Ann Thorac Surg.

[28] McAdams HP, Kirejczyk WM, Rosado-de-Christenson ML, Matsumoto S (2000). Bronchogenic cyst: imaging features with clinical and histopathologic correlation. Radiology.

[29] Durhan G, Tan AA, Duzgun SA, Akkaya S, Ariyurek OM (2020). Radiological manifestations of thoracic hydatid cysts: pulmonary and extrapulmonary findings. Insights Imaging.

[30] Jeung MY, Gasser B, Gangi A et al (2002) Imaging of cystic masses of the mediastinum. Radiographics S79-93 10.1148/radiographics.22.suppl_1.g02oc09s79 12376602

[31] Read CA, Moront M, Carangelo R, Holt RW, Richardson M (1991). Recurrent bronchogenic cyst. An argument for complete surgical excision. Arch Surg.

[32] Li L, Zeng XQ, Li YH (2010). CT-guided percutaneous large-needle aspiration and bleomycin sclerotherapy for bronchogenic cyst: report of four cases. J Vasc Interv Radiol.

[33] Schweigert M, Dubecz A, Ofner D, Stein HJ (2013). Tracheal bronchus associated with recurrent pneumonia. Ulster Med J.

[34] Keane MP, Meaney JF, Kazerooni EA, Whyte RI, Flint A, Martinez FJ (1997). Accessory cardiac bronchus presenting with haemoptysis. Thorax.

[35] McGuinness G, Naidich DP, Garay SM, Davis AL, Boyd AD, Mizrachi HH (1993). Accessory cardiac bronchus: CT features and clinical significance. Radiology.

[36] Soto-Hurtado EJ, Penuela-Ruiz L, Rivera-Sanchez I, Torres-Jimenez J (2006). Tracheal diverticulum: a review of the literature. Lung.

[37] Kurt A, Sayit AT, Ipek A, Tatar IG (2013). A multi detector computed tomography survey of tracheal diverticulum. Eurasian J Med.

[38] Polat AV, Elmali M, Aydin R, Ozbay A, Celenk C, Murat N (2014). Paratracheal air cysts: prevalence and correlation with lung diseases using multi-detector CT. J Med Imaging Radiat Oncol.

[39] Talner LB, Gmelich JT, Liebow AA, Greenspan RH (1970). The syndrome of bronchial mucocele and regional hyperinflation of the lung. Am J Roentgenol Radium Ther Nucl Med.

[40] Jennette JC, Falk RJ (1997). Small-vessel vasculitis. N Engl J Med.

[41] Webb EM, Elicker BM, Webb WR (2000). Using CT to diagnose nonneoplastic tracheal abnormalities: appearance of the tracheal wall. AJR Am J Roentgenol.

[42] Kennedy MP, Noone PG, Leigh MW (2007). High-resolution CT of patients with primary ciliary dyskinesia. AJR Am J Roentgenol.

[43] Nadel HR, Stringer DA, Levison H, Turner JA, Sturgess JM (1985). The immotile cilia syndrome: radiological manifestations. Radiology.

[44] Marini T, Hobbs SK, Chaturvedi A, Kaproth-Joslin K (2017). Beyond bronchitis: a review of the congenital and acquired abnormalities of the bronchus. Insights Imaging.

[45] Noriega Aldave AP, William SD (2014). The clinical manifestations, diagnosis and management of williams-campbell syndrome. N Am J Med Sci.

[46] Averill S, Lubner MG, Menias CO (2017). Multisystem imaging findings of cystic fibrosis in adults: recognizing typical and atypical patterns of disease. AJR Am J Roentgenol.

[47] Morrissey BM, Schock BC, Marelich GP, Cross CE (2003). Cystic fibrosis in adults: current and future management strategies. Clin Rev Allergy Immunol.

[48] Williams EA, Cox C, Chung JH, Grage RA, Rojas CA (2019). Proximal interruption of the pulmonary artery. J Thorac Imaging.

[49] Liu B, Monroe EJ, Kogut MJ (2013). Proximal interruption of the pulmonary artery: transcatheter embolization for emergent management of massive hemoptysis. Radiol Case Rep.

[50] Fiore AC, Brown JW, Weber TR, Turrentine MW. Surgical treatment of pulmonary artery sling and tracheal stenosis. Ann Thorac Surg. 2005;79(1):38–46; discussion 38–46. 10.1016/j.athoracsur.2004.06.005 15620911

[51] Hassani C, Saremi F (2017). Comprehensive cross-sectional imaging of the pulmonary veins. Radiographics.

[52] Karamlou T, Gurofsky R, Al Sukhni E (2007). Factors associated with mortality and reoperation in 377 children with total anomalous pulmonary venous connection. Circulation.

[53] Diller GP, Gatzoulis MA (2007). Pulmonary vascular disease in adults with congenital heart disease. Circulation.

[54] Berecova Z, Neuschl V, Boruta P, Masura J, Ghersin E (2012). A complex pulmonary vein varix—diagnosis with ECG gated MDCT, MRI and invasive pulmonary angiography. J Radiol Case Rep.

[55] Ferretti GR, Arbib F, Bertrand B, Coulomb M (1998). Haemoptysis associated with pulmonary varices: demonstration using computed tomographic angiography. Eur Respir J.

[56] Shah MJ, Rychik J, Fogel MA, Murphy JD, Jacobs ML (1997). Pulmonary AV malformations after superior cavopulmonary connection: resolution after inclusion of hepatic veins in the pulmonary circulation. Ann Thorac Surg.

[57] Lee KN, Lee HJ, Shin WW, Webb WR (1999). Hypoxemia and liver cirrhosis (hepatopulmonary syndrome) in eight patients: comparison of the central and peripheral pulmonary vasculature. Radiology.

[58] Braun RA, Buchmiller TL, Khankhanian N (1995). Pulmonary arteriovenous malformation complicating coccidioidal pneumonia. Ann Thorac Surg.

[59] Lee DW, White RI, Egglin TK (1997). Embolotherapy of large pulmonary arteriovenous malformations: long-term results. Ann Thorac Surg.

[60] Walker CM, Rosado-de-Christenson ML, Martinez-Jimenez S, Kunin JR, Wible BC (2015). Bronchial arteries: anatomy, function, hypertrophy, and anomalies. Radiographics.

[61] Bruzzi JF, Remy-Jardin M, Delhaye D, Teisseire A, Khalil C, Remy J (2006). Multi-detector row CT of hemoptysis. Radiographics.

[62] Yon JR, Ravenel JG (2010). Congenital bronchial artery-pulmonary artery fistula in an adult. J Comput Assist Tomogr.

[63] Woodring JH, Howard TA, Kanga JF (1994). Congenital pulmonary venolobar syndrome revisited. Radiographics.

[64] Konen E, Raviv-Zilka L, Cohen RA (2003). Congenital pulmonary venolobar syndrome: spectrum of helical CT findings with emphasis on computerized reformatting. Radiographics.

[65] Corbett HJ, Humphrey GM (2004). Pulmonary sequestration. Paediatr Respir Rev.

[66] Freedom RM, Yoo SJ, Goo HW, Mikailian H, Anderson RH (2006). The bronchopulmonary foregut malformation complex. Cardiol Young.

[67] Khushdil A, Nawaz R, Razzaq A, Ahmed N (2018). Hybrid lesion of congenital cystic adenomatoid malformation and bronchopulmonary sequestration. J Coll Physicians Surg Pak.

[68] Cass DL, Crombleholme TM, Howell LJ, Stafford PW, Ruchelli ED, Adzick NS (1997). Cystic lung lesions with systemic arterial blood supply: a hybrid of congenital cystic adenomatoid malformation and bronchopulmonary sequestration. J Pediatr Surg.

[69] Masoomi MA, Al-Kandari L, Al-Shammeri I. Combining 3D rendering model and pixelated quantitative CT for rapid assessment of lung affected with COVID-19 and need for oxygenation. BMJ Case Rep. 2021;14(1). 10.1136/bcr-2020-241060 PMC7849877 33514617

[70] Ley-Zaporozhan J, Ley S, Weinheimer O (2008). Quantitative analysis of emphysema in 3D using MDCT: influence of different reconstruction algorithms. Eur J Radiol.

[71] Kang M-J, Park CM, Lee C-H, Goo JM, Lee HJ (2010). Dual-energy CT: clinical applications in various pulmonary diseases. Radiographics.

[72] Afadzi M, Lysvik EK, Andersen HK, Martinsen ACT (2019). Ultra-low dose chest computed tomography: effect of iterative reconstruction levels on image quality. Eur J Radiol.

[73] Ebner L, Knobloch F, Huber A (2014). Feasible dose reduction in routine chest computed tomography maintaining constant image quality using the last three scanner generations: from filtered back projection to sinogram-affirmed iterative reconstruction and impact of the novel fully integrated detector design minimizing electronic noise. J Clin Imaging Sci.

[74] Edelman RR, Koktzoglou I (2019). Noncontrast MR angiography: an update. J Magn Reson Imaging.

[75] Xu J, McGorty KA, Lim RP (2012). Single breathhold noncontrast thoracic MRA using highly accelerated parallel imaging with a 32-element coil array. J Magn Reson Imaging.

[76] Togao O, Tsuji R, Ohno Y, Dimitrov I, Takahashi M (2010). Ultrashort echo time (UTE) MRI of the lung: assessment of tissue density in the lung parenchyma. Magn Reson Med.

[77] Heidenreich JF, Weng AM, Metz C (2020). Three-dimensional ultrashort echo time MRI for functional lung imaging in cystic fibrosis. Radiology.

[78] Ohno Y, Koyama H, Yoshikawa T (2016). Pulmonary high-resolution ultrashort TE MR imaging: Comparison with thin-section standard- and low-dose computed tomography for the assessment of pulmonary parenchyma diseases. J Magn Reson Imaging.

[79] Voskrebenzev A, Vogel-Claussen J (2021). Proton MRI of the lung: how to tame scarce protons and fast signal decay. J Magn Reson Imaging.

[80] Bauman G, Eichinger M (2012). Ventilation and perfusion magnetic resonance imaging of the lung. Pol J Radiol.

[81] Berthezene Y, Vexler V, Clement O, Muhler A, Moseley ME, Brasch RC (1992). Contrast-enhanced MR imaging of the lung: assessments of ventilation and perfusion. Radiology.

[82] Ley S, Ley-Zaporozhan J (2012). Pulmonary perfusion imaging using MRI: clinical application. Insights Imaging.

[83] Ohno Y, Chen Q, Hatabu H (2001). Oxygen-enhanced magnetic resonance ventilation imaging of lung. Eur J Radiol.

[84] Hatabu H, Tadamura E, Chen Q (2001). Pulmonary ventilation: dynamic MRI with inhalation of molecular oxygen. Eur J Radiol.

[85] Arai TJ, Prisk GK, Holverda S et al (2011) Magnetic resonance imaging quantification of pulmonary perfusion using calibrated arterial spin labeling. J Vis Exp 2011(51) 10.3791/2712 PMC3197117 21673635

[86] Bolar DS, Levin DL, Hopkins SR (2006). Quantification of regional pulmonary blood flow using ASL-FAIRER. Magn Reson Med.

[87] Bondesson D, Schneider MJ, Gaass T (2019). Nonuniform Fourier-decomposition MRI for ventilation- and perfusion-weighted imaging of the lung. Magn Reson Med.

[88] Klimeš F, Voskrebenzev A, Gutberlet M (2021). 3D phase-resolved functional lung ventilation MR imaging in healthy volunteers and patients with chronic pulmonary disease. Magn Reson Med.

[89] Behrendt L, Voskrebenzev A, Klimeš F (2020). Validation of automated perfusion-weighted phase-resolved functional lung (PREFUL)-MRI in Patients With Pulmonary Diseases. J Magn Reson Imaging.

